# Biochemical and transcript level differences between the three human phosphofructokinases show optimisation of each isoform for specific metabolic niches

**DOI:** 10.1042/BCJ20200656

**Published:** 2020-11-27

**Authors:** Peter M. Fernandes, James Kinkead, Iain McNae, Paul A.M. Michels, Malcolm D. Walkinshaw

**Affiliations:** School of Biological Sciences, University of Edinburgh, Michael Swann Building, Max Born Crescent, Edinburgh EH9 3BF, U.K.

**Keywords:** enzyme kinetics, human, liver metabolism, muscle metabolism, phosphofructokinase, platelet

## Abstract

6-Phosphofructokinase-1-kinase (PFK) tetramers catalyse the phosphorylation of fructose 6-phosphate (F6P) to fructose 1,6-bisphosphate (F16BP). Vertebrates have three PFK isoforms (PFK-M, PFK-L, and PFK-P). This study is the first to compare the kinetics, structures, and transcript levels of recombinant human PFK isoforms. Under the conditions tested PFK-M has the highest affinities for F6P and ATP (K_0.5_^ATP^ 152 µM; K_0.5_^F6P^ 147 µM), PFK-P the lowest affinities (K_0.5_^ATP^ 276 µM; K_0.5_^F6P^ 1333 µM), and PFK-L demonstrates a mixed picture of high ATP affinity and low F6P affinity (K_0.5_^ATP^ 160 µM; K_0.5_^F6P^ 1360 µM). PFK-M is more resistant to ATP inhibition compared with PFK-L and PFK-P (respectively, 23%, 31%, 50% decreases in specificity constants). GTP is an alternate phospho donor. Interface 2, which regulates the inactive dimer to active tetramer equilibrium, differs between isoforms, resulting in varying tetrameric stability. Under the conditions tested PFK-M is less sensitive to fructose 2,6-bisphosphate (F26BP) allosteric modulation than PFK-L or PFK-P (allosteric constants [K_0.5_^ATP+F26BP^/K_0.5_^ATP^] 1.10, 0.92, 0.54, respectively). Structural analysis of two allosteric sites reveals one may be specialised for AMP/ADP and the other for smaller/flexible regulators (citrate or phosphoenolpyruvate). Correlations between PFK-L and PFK-P transcript levels indicate that simultaneous expression may expand metabolic capacity for F16BP production whilst preserving regulatory capabilities. Analysis of cancer samples reveals intriguing parallels between PFK-P and PKM2 (pyruvate kinase M2), and simultaneous increases in PFK-P and PFKFB3 (responsible for F26BP production) transcript levels, suggesting prioritisation of metabolic flexibility in cancers. Our results describe the kinetic and transcript level differences between the three PFK isoforms, explaining how each isoform may be optimised for distinct roles.

## Introduction

6-Phosphofructokinase-1-kinase (PFK) (EC 2.7.1.11) catalyses the phosphorylation of fructose 6-phosphate (F6P) to fructose 1,6-bisphosphate (F16BP). PFK regulation is complex, befitting its role as the first committed step of glycolysis. The extraordinarily ancient nature of this enzyme is reflected in the melange of structural, kinetic, and regulatory properties found in PFKs of different species [1]. Vertebrate PFK monomers are approximately twice the size of bacterial counterparts, suggesting a eukaryotic gene duplication and fusion process [2]. PFK gene duplication appears to have occurred twice in certain eukaryotic lineages, even before the separation of the fungi [3], with vertebrate genomes possessing three separate PFK genes, historically labelled as PFK-M (muscle), PFK-L (liver), and PFK-P (platelet). With the exception of skeletal muscle, which only expresses PFK-M, human tissues express all three PFK isoforms, though in varying ratios [4]; exact quantification of differential PFK expression in each tissue has not been attempted. In this paper we provide a detailed comparison of the kinetic properties of each human isoform, placing this into the context of sequence and structural differences, as well as isoform-specific transcript level changes between human tissues.

**Figure 1.**
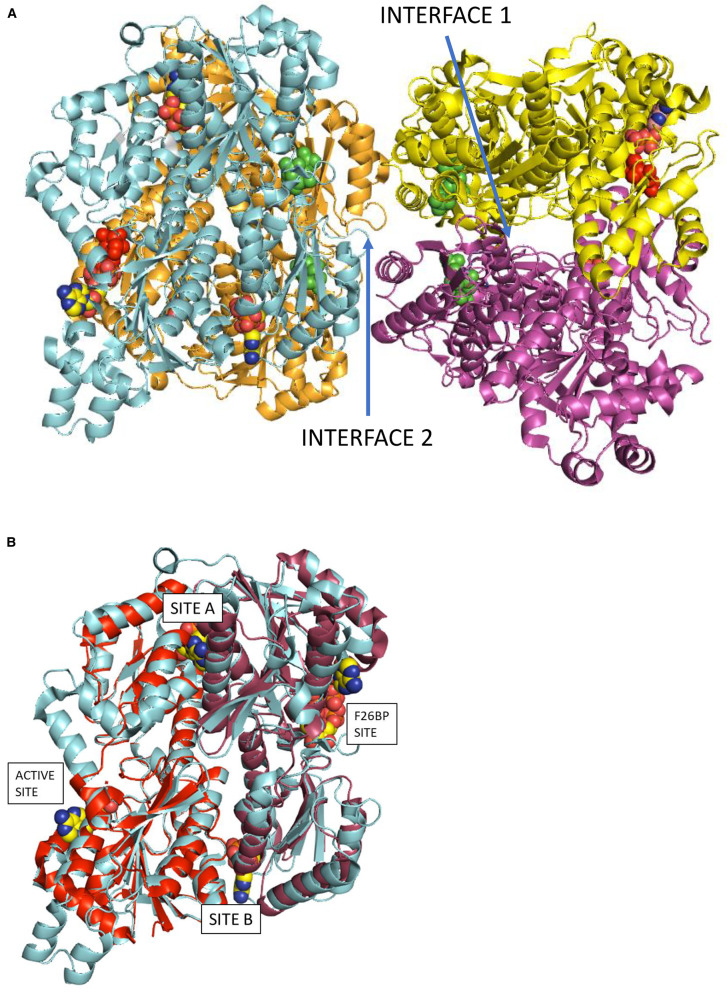
Models of PFK structures showing chain interface sites and allosteric binding sites. Panel **A** shows the PFK-P tetrameric structure (PDB reference: 4XZ2) with the locations of the active sites and allosteric sites indicated. Interface residues between protomer pairs were identified using PDBePISA. Interface 1 is between chains A (orange) and B (cyan) (or C (purple) and D (yellow)). The view is along a pseudo 2-fold symmetry axis of the B/A dimer. There is an equivalent symmetry related ‘interface 1’ between chains C and D. Interface 2 is between chains B and C (or the symmetry related chains A and D). The active site of chain B shows F6P (red) and ADP (atom-type colour). The 4 molecules of F26BP in green are close to Interface 2. Two ADP molecules (atom-type colour) are modelled into the remaining two allosteric sites of chain B (cyan) labelled in Panel **B** as site A and site B. Panel **B** shows an overlay of EcPFK (PDB reference: 1PFK) and PFK-P (cyan). Chain A of EcPFK (1PFK) fitted onto residues 1–384 of chain B of PFK-P is red and chain B of EcPFK (1PFK) fitted onto residues 385–784 of chain B of PFK-P is raspberry. The allosteric binding sites A and B are occupied by ADP from the EcPFK X-ray structure.

Mammalian PFKs are inactive as monomers and dimers, but active as tetramers [5]; heteromeric tetramers (comprising more than one isoform) can form but their biological significance remains unknown [6]. Additionally, PFK-L can adopt a multimeric filamentous form [7]. PFK paralogues are remarkably similar in mass and polypeptide length despite the hundreds of millions of years of evolution separating each isoform (Table 1). A protein crystal structure of human PFK-P is available [8]; crystal structures for full length PFK-L and PFK-M have yet to be published, though given the high levels of amino acid identity the architecture of all three isoforms is likely to be conserved (Figure 1). The well characterised *Escherichia coli* PFK (EcPFK) and *Trypanosoma brucei* PFK (TbPFK) crystal structures (PDB references 1PFK and 6SY7, respectively) possess a single active site and a further, single, allosteric site per monomer: gene duplication and fusion in eukaryotes therefore suggests mammalian PFKs should possess two active sites. However, analysis of published human PFK structures indicate conversion of the active site of one of the duplicated genes into an allosteric site that binds fructose 2,6-bisphosphate (F26BP) which acts as an allosteric activator. There is also a degree of sequence conservation around the ADP or AMP allosteric sites observed in the EcPFK and TbPFK X-ray structures, with phosphate binding observed in the N-terminal and C-terminal domains of each mammalian PFK protomer meaning that mammalian PFK tetramers putatively contain 4 active sites and 12 allosteric sites (Figure 1A).

**Table 1:** Comparison of human PFK-M, PFK-L and PFK-P sequences shows weak conservation at Interface 2 residues compared with Interface 1 residues

			PFK-M	PFK-L	PFK-P
**PFK-M**		Overall identity	-	69%	68%
-	780 AA	Interface1	-	88% (58/66)	91% (60/66)
-	85.18 kDa	Interface 2	-	63% (12/19)	79% (15/19)
		ATP site	-	88% (7/8)	100% (8/8)
		F26BP site		84% (16/19)	95% (18/19)
		Allosteric site A	-	87% (13/15)	80% (12/15)
		Allosteric site B	-	88% (15/17)	82% (14/17)
**PFK-L**		Overall identity	69%	-	71%
-	780 AA	Interface1	88% (58/66)	-	89% (59/66)
-	85.02 kDa	Interface 2	63% (12/19)	-	58% (11/19)
		ATP site	88% (7/8)	-	88% (7/8)
		F26BP site	84% (16/19)	-	84% (16/19)
		Allosteric site A	87% (13/15)	-	67% (10/15)
		Allosteric site B	76% (13/17)	-	82% (14/17)
**PFK-P**		Overall identity	68%	71%	-
-	784 AA	Interface1	91% (60/66)	89% (59/66)	-
-	85.60 kDa	Interface 2	79% (15/19)	58% (11/19)	-
		ATP site	100% (8/8)	88% (8/8)	-
		F26BP site	95% (18/19)	84% (16/19)	-
		Allosteric site A	80% (12/15)	67% (10/15)	-
		Allosteric site B	82% (14/17)	88% (15/17)	-

Residues involved in Interface 1, Interface 2, and binding pockets for ATP and F26BP (Figure 1) were identified using PDBePISA^1^ using the PFK-P structure (PDB 4XYJ). Allosteric sites A and B (Figure 1) were identified by overlying the structure of EcPFK complex with ADP (PDB PFK1). Supplementary Table S2 compares Interface 1 amino acid sequences; Supplementary Table S3 compares Interface 2 amino acid sequences.

Human PFK activity is tightly controlled through multiple processes, including allosteric regulation. The most potent natural activator is F26BP, which has no other known function in mammalian cells except inhibition of the gluconeogenic enzyme fructose-1,6-bisphosphatase (FBPase-1). F26BP production is highly controlled through the bi-functional enzyme PFK-2 (also known as FBPase-2, fructose bisphosphatase-2, 6-phosphofructo-2-kinase/fructose-2,6-bisphosphatase) comprising numerous isoforms resulting from four genes and multiple splice variants [9]. The isoform PFKFB3 favours the formation of F26BP and is an anti-cancer drug target [10]. Aside from F26BP, the major activators of mammalian PFK are AMP and ADP, with the major inhibitors being ATP, citrate, lactate, and acyl-CoA [11]. Multiple other modulators of PFK activity have been described, including several commonly used drugs (Supplementary Table S1) and pH [12–15]. PFK activity can also be altered by changes in the propensity of inactive monomers/dimers to coalescence into active tetramers. Multiple influencers of oligomerisation status have been described, including cancer-associated mutations [8], redox status [14], and allosteric effectors [15]. Post-translational modifications regulate PFK activity; for example, O-GlcNAc modification of Ser529 prevents F26BP binding, enhancing tumour cell survival in hypoxic conditions [16]. Formation of metabolons (complexes of metabolic enzymes) also change PFK activity by enabling channelling of glycolytic intermediates, ameliorating the limiting effects of diffusion, and allowing PFK dimers to become active [17]. The regulatory differences between PFK isoforms, particularly with regard to allosteric modulators, have not been systematically explored until now, despite the increasing evidence linking disturbances in glycolysis with diseases including Alzheimer's disease [18], multiple sclerosis [19], and cancer [20]. Previous studies have focussed on a single isoform, or at the most two isoforms, and true comparative studies of the three isoforms are rare, often using partially purified tissue-derived enzymes (Supplementary Table S10) [4,21,22]. Our results, which systematically characterise the differences between human PFK isoforms in terms of kinetic properties, sequence and structural changes, and tissue-specific transcript levels, help explain the different and specific roles played by each of the three PFK isoforms.

## Methods

### Production of human phosphofructokinases

Recombinant human PFKs were expressed in PFK-deficient *Saccharomyces cerevisiae* and purified using the methods described previously [23], adapting earlier materials and protocols [24]. Production of PFK intended for use in ATP titration experiments used 1 mM F6P as a tetramer stabilising agent in protein purification and storage buffers. Production of PFK intended for use in F6P titration experiments used 1 mM ATP as a tetramer stabilising agent in protein purification and storage buffers. Experimental details on the purification and characterisation procedures including figures showing SDS–PAGE gels of the highly pure PFKs used in the kinetic experiments described in this work have been recently published [23]. Western blots, and MALDI-TOF mass spectrometry via in-gel and in-solution digests showed no evidence of N-linked glycosylation (data not shown) and addressed concerns over possible inappropriate glycosylation from the yeast expression system, which may interfere with enzyme activity [16].

### Determination of kinetic characteristics using ALDO-TIM-G3PDH enzyme-linked kinetic assay

Assay buffer consisted of 50 mM TEA, 100 mM KCl, 10 mM MgCl_2_, 1 mM Tris(2-carboxyethyl)phosphine hydrochloride (TCEP), 10% glycerol, pH 7.4. Assay mix consisted of 5 units/ml glycerol-3-phosphate dehydrogenase (G3PDH, Sigma–Aldrich G6880), 2.5 units/ml aldolase (Sigma–Aldrich A2714), 25 units/ml triosephosphate isomerase (TIM, Sigma–Aldrich T6258), and 1.25 mM NADH. ATP and F6P were obtained from Sigma–Aldrich (A2383 and F3627, respectively). For ATP titrations, 38 µl of buffer or ligand were added to a clear non-binding 96-well plate. 40 µl of assay mix containing 10 mM F6P (final concentration 4 mM) was added and the plate was incubated at 25°C for 5 min. An amount of 2 µl of PFK isoforms were added to each well from stock concentrations of 150 µg/ml and the reaction was immediately started with 20 µl of ATP at varying concentrations. For F6P titrations, 30 µl of buffer or ligand was added to a clear non-binding 96-well plate. An amount of 40 µl of assay mix containing 1.25 mM ATP (final concentration 0.5 mM) was added and the plate was incubated at 25°C for 5 min. An amount of 5 µl of PFK isoforms were added to each well from stock concentrations of 200 µg/ml and the reaction was immediately started with 20 µl F6P at varying concentrations. All ligands and substrates (including ATP) were used at pH 7.4. All metabolites were obtained from Sigma–Aldrich unless otherwise stated. Ligand concentrations were chosen based on normal physiological concentrations (Supplementary Table S2) and ability to demonstrate effects above the signal-to-noise thresholds for the enzyme-linked assay. A SpectraMax M5 Multi-Mode Microplate Reader was used to measure A_340nm_ at 13 s intervals for 10 min. All experiments were performed in triplicate unless otherwise stated.

Time-dependent absorbance change was converted into rate of NADH oxidation (µM s^−1^) or specific activity (µmol min^−1^ mg protein^−1^) using the Beer-Lambert law (molar extinction coefficient of NADH 6.22 mM^−1^ cm^−1^). The initial rate of reaction was used to avoid accumulation of ADP, which activates PFK, interfering with the determination of kinetic properties. Non-linear regression analysis was performed on substrate titration data using GraphPad Prism 7, with curves fitted using allosteric sigmoidal models, enabling determination of steady-state kinetic values.

### Sequence and structure analysis

Output from PISA [25] using the coordinates from human PFK-P (PDB code 4xz2 [26]) was used to determine those residues involved in interchain contacts across Interface 1 and Interface 2 and the binding pockets for ATP and F26BP. All residues contributing a Buried Surface Area (BSA) value greater than 0.5 Å^2^ were selected and compared with the equivalent residues in PFK-M and PFK-L

### FANTOM5 data

The FANTOM5 database was interrogated for each of the enzymes of interest using the ZENBU visualisation system. Phase 1 and 2 FANTOM5 data were used, containing hg19 assembly (Genome Reference Consortium 2009). All data used were RLE normalised and the robust dataset was preferred. The gene of interest was selected and data in the direction of PFK gene transcription were examined, excluding time-course experimental data. Tissue-specific data were identified from the appropriate promoters in the ‘FANTOM5 PHASE 1and2 FREEZE DPI clusters human (robust set with expression)’ channel. Transcripts per million (TPM) scores were identified for each sample, with samples categorised manually into tissue types and mean TPM scores calculated. Pearson correlation coefficients of transcription were calculated for pairwise comparisons of PFK isoform with other metabolic enzymes.

## Results and discussion

### Biochemical comparison of human PFK isoforms

#### Kinetic parameters for PFK isoforms

**Figure 2.**
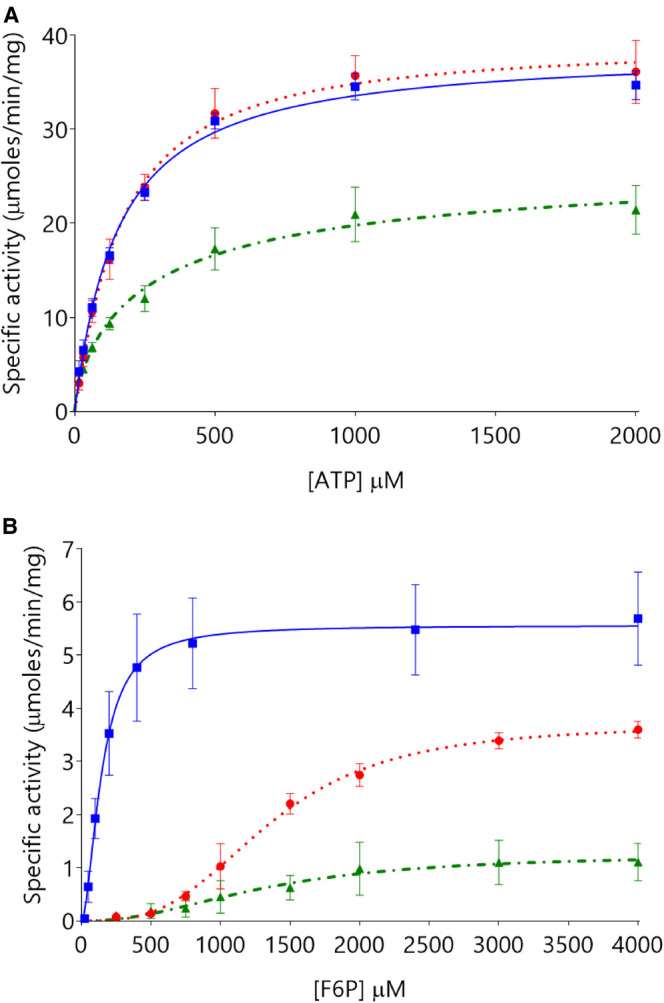
PFK-L is as active as PFK-M with respect to ATP, but much less active with respect to F6P. Panel **A** shows similar kinetic properties with respect to ATP titrations (F6P 4 mM) for PFK-M (blue squares 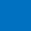 with solid lines) and PFK-L (red circles 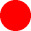 with dotted lines) with PFK-P (green triangles 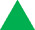 with dot-dash lines) showing lower activity. Panel **B** shows PFK-M to be much more active than PFK-L or PFK-P with respect to F6P titrations (ATP 0.5 mM). (*n* = 6).

PFK kinetic parameters values from all three human PFK isoforms were determined for ATP and F6P (Table 2), derived from activity versus substrate concentration plots (Figure 2). PFK-M has high affinity for F6P and ATP (K_0.5_^F6P^ 147 µM; K_0.5_^ATP^ 152 µM); PFK-L has low affinity for F6P but high affinity for ATP and F6P (K_0.5_^F6P^ 1360 µM; K_0.5_^ATP^ 160 µM); PFK-P has low affinity for F6P and ATP (K_0.5_^F6P^ 1333 µM; K_0.5_^ATP^ 276 µM). Cooperativity, as measured by Hill plot values, was absent for ATP titrations but present for F6P titrations: PFK-L shows the most cooperativity (3.10), followed by PFK-P (2.18), and PFK-M the least (1.82). Specificity constants (*k*_cat_^substrate^/K_0.5_^substrate^), a composite value amalgamating maximal activity and substrate affinity to measure catalytic efficiency, showed PFK-M to be most efficient, followed by PFK-L, and then PFK-P (*k*_cat_^F6P^/K_0.5_^F6P^ 0.04, 0.002, 0.0009 s^−1^ µM^−1^, respectively; *k*_cat_^ATP^/K_0.5_^ATP^ 0.37, 0.36, 0.14 s^−1^ µM^−1^, respectively). Differences in maximal activity between F6P and ATP titrations are likely caused by changes in the order that substrates are added to the assay mix (see Methods), as occurs in trypanosomatid PFKs [27]. Prolonged incubation with F6P may favour the active tetramer form compared with incubation with ATP. These data are valid for the active tetrameric enzyme but it is known that PFK-L can form higher-order oligomers, including a filamentous form [7]. These higher-order PFK-L oligomers may have significantly different kinetic properties with increased affinity for F6P [28]. Similar effects are seen in the presence of PEG [29].

**Table 2:** Human PFK isoforms display distinct kinetic properties

		PFK-M	PFK-L	PFK-P
**F6P**	V_max_^F6P^ (µmoles/min.mg)	5.56 (0.18)	3.69 (0.09)	1.26 (0.21)
	K_0.5_^F6P^ (µM)	147.1 (13.6)	1360 (40)	1333 (312)
	*k*_cat_^F6P^ (s^−1^)	8.11 (0.27)	5.38 (0.14)	1.83 (0.31)
	*k*_cat_^F6P^/K_0.5_^F6P^ (s^−1^ µM^−1^)	3.8 × 10^−2^ (1.32 × 10^−2^)	2.71 × 10^−3^ (2.25 × 10^−3^)	9.45 × 10^−4^ (6.73 × 10^−4^)
	h (F6P)	1.82 (0.26)	3.10 (0.22)	2.18 (0.75)
**ATP**	V_max_^ATP^ (µmoles/min.mg)	38.4 (0.9)	39.3 (1.2)	27.2 (2.5)
	K_0.5_^ATP^ (µM)	151.8 (10.0)	160.1 (13.5)	275.6 (84.4)
	k_cat_^ATP^ (s^−1^)	56.0 (1.2)	57.3 (1.7)	39.7 (1.7)
	k_cat_^ATP^/K_0.5_^ATP^ (s^−1^ µM^−1^)	0.37 (0.12)	0.36 (0.13)	0.14 (0.02)
	h (ATP)	1.03 (0.05)	1.12 (0.08)	0.76 (0.09)
**GTP**	V_max_^GTP^ (µmoles/min.mg)	17.82 (0.44)	25.39 (0.56)	12.30 (0.62)
	K_0.5_^GTP^ (µM)	96.7 (8.0)	157.8 (10.8)	131 (20.9)
	*k*_cat_^GTP^ (s^−1^)	26.0 (0.6)	37.0 (0.8)	17.9 (0.9)
	*k*_cat_^GTP^/K_0.5_^GTP^ (s^−1^ µM^−1^)	0.27 (0.08)	0.23 (0.07)	0.14 (0.04)
	h (GTP)	1.19 (0.12)	1.18 (0.08)	1.12 (0.18)

Values are for kinetic properties determined by titrating the substrate listed in first column. For F6P titrations: ATP = 0.5 mM; *n* = 6. For ATP titrations: F6P = 4 mM; *n* = 6. For GTP titrations: F6P = 4 mM; *n* = 3. Values are mean averages with standard deviations in parentheses.

**Figure 3.**
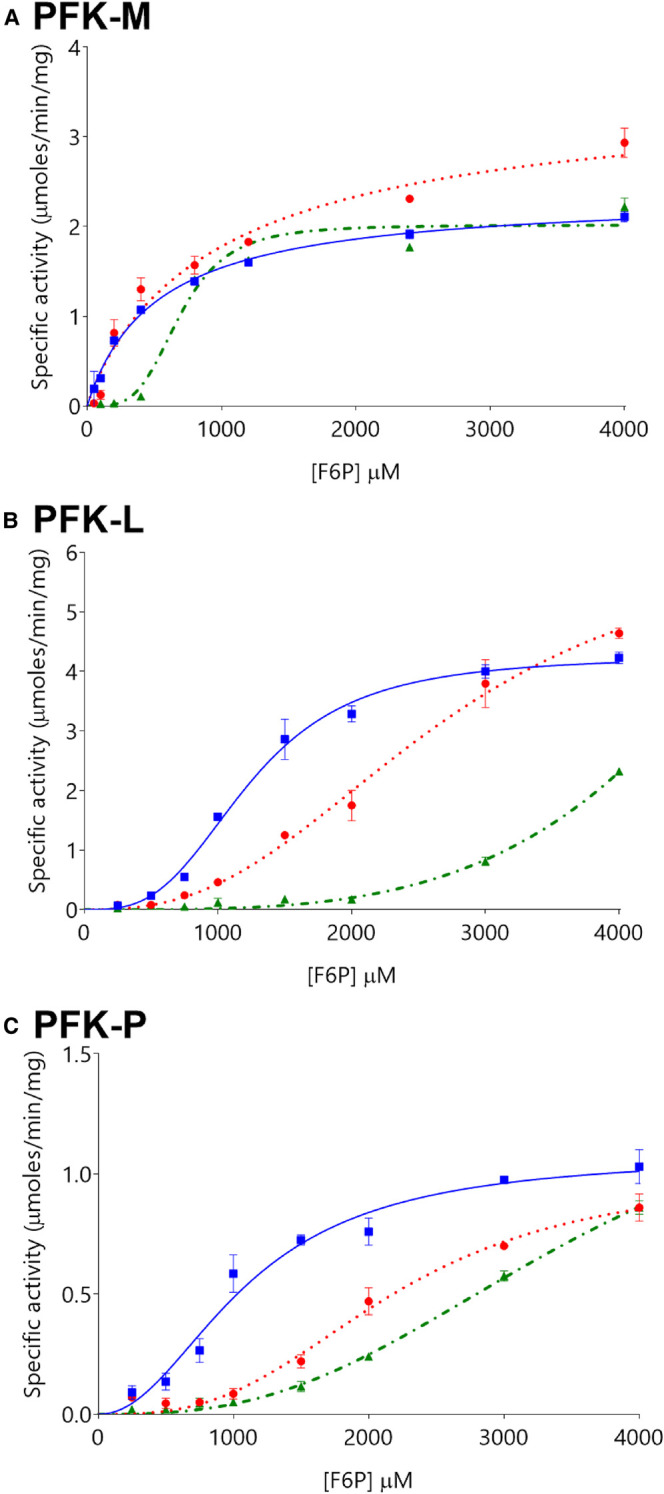
The inhibitory effect of ATP differs between the three PFK isoforms. Levels of ATP inhibition differ between PFK-M (panel **A**), PFK-L (panel **B**), and PFK-P (panel **C**), at ATP concentrations of 0.5 mM (blue squares 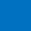 with solid lines), 1 mM (red circles 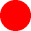 with dotted lines), and 2 mM (green triangles 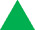 with dot-dash lines). (*n* = 3). Note that *y*-axis scales differ between Panels.

Titration of ATP concentrations (Figure 3), resulted in an initial increase in enzymatic activity with increasing ATP concentrations before decreasing at higher ATP concentrations. Thus, the specific activity of PFK-M increases 23% as ATP concentration increases from 0.25 mM to 0.5 mM ATP, but then decreases 21% as the ATP concentration increases further from 0.5 mM to 1 mM ATP. Comparing the specificity constants for each isoform at 0.5 mM and 1 mM ATP demonstrates that PFK-M is most resistant to ATP inhibition (21% decrease at 1 mM ATP), PFK-L is intermediate (31% decrease), and PFK-P is most susceptible (50% decrease). This hierarchy is the same as described previously in the literature [21,30]. High F6P concentrations relieved the inhibitory effects of ATP, though to differing levels for each PFK isoform (Figure 3). Derived kinetic parameters were highly dependent on experimental timings, in keeping with time and concentration-dependent enzyme deactivation. This interpretation is valid for the conditions tested (pH 7.4) but may not hold true at other pH values, with previous studies showing PFK-M activity being highly dependent on pH [12,13]. This caveat is particularly important given muscle pH varies depending on time and intensity of muscle activity [31]. Results from previous studies are shown in Supplementary Table S10A (K_m_^F6P^) and 10B (K_m_^ATP^); these indicate broadly similar absolute results but differing hierarchies of activity for each isoform, which may relate to differences in tissue purification and assay conditions, particularly regarding ATP, ADP, and other ligand concentrations.

Interestingly, we show that GTP is an alternative phospho donor for all three PFK isoforms, though resulting in lower maximal velocity (Table 2). Previous studies have demonstrated binding of GTP to rabbit muscle PFK, though kinetic data were lacking [32,33]. K_0.5_^GTP^ values showed each PFK isoform to have higher affinities for GTP than ATP (PFK-M: 97 µM vs. 152 µM; PFK-L: 158 µM vs. 160 µM; PFK-P: 131 µM vs. 276 µM), though comparison of specificity constants indicate that ATP is a more efficient substrate than GTP (0.37 vs. 0.27 s^−1^ µM^−1^; 0.36 vs. 0.23 s^−1^ µM^−1^; 0.14 vs. 0.14 s^−1^ µM^−1^, respectively). This raises the question of whether GTP is a physiologically relevant substrate. The difference in intra-cellular GTP and ATP concentrations (0.3 mM and 2.1 mM, respectively [34]), implies that PFK has a considerably greater capacity to sustain glycolytic flux using ATP rather than GTP. Thus, under normal conditions, GTP is predicted to be less important than ATP, except in situations where GTP concentrations might exceed ATP concentrations.

#### Comparison of the amino acid sequences of the interface regions of the human isoforms

We rationalised differences in kinetic behaviour by carrying out a detailed sequence comparison of the three isoforms using the template provided by the X-ray structure of PFK-P (PDB 4xz2). The active tetramer is formed by a dimer of dimers (Figure 1) with the A–B and C–D dimers held together by ‘Interface 1’ which has ∼40 hydrogen bonds including nine salt bridges. Each chain contributes an average buried surface area of ca. 2160 Å^2^ to this interface. The 66 residues that contribute more than 4 Å^2^ towards the buried surface area of interface 1 are listed in Supplementary Table S3 and compared with equivalent sequences for PFK-L and PFK-M. PFK-P differs from PFK-M by 6/66 residues (I90V, V91I, D448E being conservative, and A677S, S571A, I476K not being conservative) and from PFK-L by 7/66 residues. PFK-L differs from PFK-M by 8/66 residues.

A similar analysis for Interface 2 shows that D–A (and B–C) interactions for PFK-P are stabilised by seven hydrogen bonds incorporating four salt bridges, comprising interactions from 19 residues in total. Each chain contributes an average of ca. 700 Å^2^ to this interface. Interestingly, it is this smaller interface that is conserved in the dimeric X-ray structure of rabbit PFK-M that overlays very well with the human PFK-P structure (RMS fits of 0.9 Å for monomer and 1.3 Å for dimer). The 19 residues that contribute more than 4 Å^2^ towards the buried surface area of interface 2 are listed in Supplementary Table S4 and compared with equivalent sequences in the PFK-L and PFK-M isoforms. There are 11/19 (58%) of residues conserved between PFK-P and PFK-L, and 15/19 (79%) residues conserved between PFK-P and PFK-M at this interface. As discussed below, this interface regulates the equilibrium between inactive dimer and active tetramer and is therefore key to understanding the differences in enzyme activity and allosteric behaviour of the isoforms.

#### Allosteric modulation of PFK activity by F26BP

**Figure 4.**
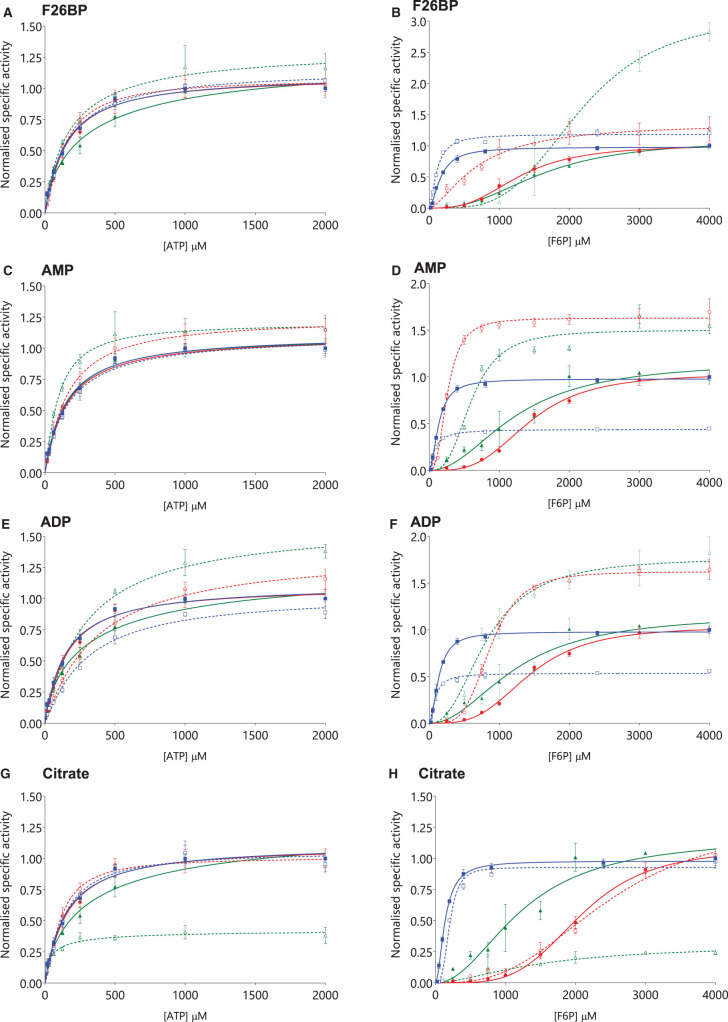
PFK-P activity is modulated more by allosteric regulators than PFK-M or PFK-L. The effects of F26BP (10 µM) on ATP titrations (**A**) are less pronounced than on F6P titrations (**B**), as is found for AMP (1200 µM) on ATP (**C**) and F6P (**D**). ADP (600 µM) inhibits PFK-M and activates PFK-L and PFK-P for ATP titrations (**E**) and F6P titrations (**F**). Citrate (600 µM) inhibits PFK-P for ATP titrations (**G**) and F6P titrations (**H**). PFK-M (blue squares 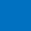 with solid blue lines), PFK-L (red circles 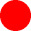 with solid red lines), PFK-P (green triangles 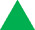 with solid green lines). Curves obtained in the presence of allosteric modulators are given in equivalent dashed lines with open symbols. (*n* = 3, all values normalised to highest respective control value).

F26BP strongly activated all three isoforms under the conditions tested, with AC_50_^F26BP^ values showing that PFK-L was most sensitive (0.17 µM ± 0.16), followed by PFK-M (6.27 µM ± 1.57) and PFK-P (9.13 µM ± 2.19) (Supplementary Table S5). F26BP strikingly activates PFK-L at low to medium (physiological) concentrations of F6P, and PFK-P at high concentrations of F6P (between 1 mM and 2 mM), with PFK-M showing a more modest activation. In contrast, F26BP has little effect on kinetic properties for ATP titrations, except for PFK-P, where the K_0.5_^ATP^ almost halves (Figure 4, Table 3, Supplementary Table S5). Thus, for ATP PFK-M is less sensitive to F26BP allosteric modulation than PFK-L or PFK-P (allosteric constants [K_0.5_^ATP+F26BP^/K_0.5_^ATP^] 1.10, 0.92, 0.54, respectively). The use of coupled-enzyme assays is limited by potential effects of ligands on auxiliary enzymes and accumulation of active metabolites (such as ADP in our assay). These effects were ameliorated by the use of auxiliary enzyme in excess concentrations, and the lack of known allosteric effects of F26BP on any auxiliary enzymes, as well as the use of the initial rate of reaction to calculate kinetic properties. Of the two previous comparative studies to investigate the effects of F26BP, both show a dramatic increase in F6P affinities in the presence of F26BP, though there are differences in absolute values reported and the relative responses of each isoform in comparison with other isoforms [4,21]. An isoform-specific comparison of how affinity for ATP changes in the presence of F26BP has not been previously published.

**Table 3:** Summary of effects of various modulators of PFK activity on each human PFK isoform

Metabolite		PFK-M	PFK-L	PFK-P
AMP (1200 µM)	*k*_cat_^ATP^/K_0.5_^ATP^ (s^−1^ µM^−1^)	−12%	+9%	+220%
	*k*_cat_^F6P^/K_0.5_^F6P^ (s^−1^ µM^−1^)	−25%	+766%	+162%
ADP (600 µM)	*k*_cat_^ATP^/K_0.5_^ATP^ (s^−1^ µM^−1^)	−52%	−47%	+39%
	*k*_cat_^F6P^/K_0.5_^F6P^ (s^−1^ µM^−1^)	−13%	+157%	+129%
GDP (1100 µM)	*k*_cat_^ATP^/K_0.5_^ATP^ (s^−1^ µM^−1^)	+4.3%	−48%	−0.2%
	*k*_cat_^F6P^/K_0.5_^F6P^ (s^−1^ µM^−1^)	ND	ND	ND
F26BP (10 µM)	*k*_cat_^ATP^/K_0.5_^ATP^ (s^−1^ µM^−1^)	−7.4%	+6.5%	+88%
	*k*_cat_^F6P^/K_0.5_^F6P^ (s^−1^ µM^−1^)	+82%	+148%	+111%
Citrate (600 µM)	*k*_cat_^ATP^/K_0.5_^ATP^ (s^−1^ µM^−1^)	+5%	+28%	+136%
	*k*_cat_^F6P^/K_0.5_^F6P^ (s^−1^ µM^−1^)	−36%	+2%	−78%
PEP (1100 µM)	*k*_cat_^ATP^/K_0.5_^ATP^ (s^−1^ µM^−1^)	−11%	+1%	−40%
	*k*_cat_^F6P^/K_0.5_^F6P^ (s^−1^ µM^−1^)	−7%	+92%	+9%

Effect of modulators with respect to ATP and F6P, as measured by percentage changes in specificity constants compared with control PFK without modulator (absolute values in Table 2).

Supplementary Table S6 compares the F26BP binding site in the three human isoforms. The F26BP pocket is well conserved; out of the 19 residues that interact with F26BP, there is only one substitution (G584A) between PFK-M and PFK-P. There are 3/19 differences between PFK-L and PFK-P isoforms (G584T, M583V and V539I), which may help explain the >50-fold decrease in AC_50_^F26BP^ for PFK-L compared with PFK-P. Figure 4B shows that PFK-L has a dramatic increase in activity at physiological concentrations of F6P when low concentrations of F26BP are present, whereas PFK-P only shows a response at supra-physiological concentrations of F6P. This difference is postulated to be due to the differences in AC_50_^F26BP^ between PFK-L and PFK-P. Previous analyses have shown that bound F26BP acts as a bridge to lock and tighten binding across the A/B (and C/D) interfaces [35]. Nineteen residues binding F26BP were identified, with full conservation of the 5 residues (26%) involved in interactions across Interface 1 (Supplementary Table S6).

#### Allosteric modulation of PFK activity by AMP and ADP

With respect to ATP titrations, AMP has minimal effect on PFK-M, a modest effect on PFK-L, but causes a marked increase in maximal activity and ATP affinity for PFK-P (Figure 4). For F6P titrations the effects are even more striking: PFK-L is very strongly activated by AMP (more than 5-fold decrease in K_0.5_^F6P^), PFK-P is also activated (two-fold decrease in K_0.5_^F6P^), but the near two-fold decrease in K_0.5_^F6P^ for PFK-M is offset by a concomitant reduction in V_max_^F6P^ leading to an overall decrease in catalytic efficiency (Figure 4, Table 3, Supplementary Table S5). The effect of ADP is more complicated: inhibiting PFK-M, having mixed effects on PFK-L, and activating PFK-P (Figure 4, Table 3, Supplementary Table S5). ADP markedly increases affinity for F6P and *V*_*max*_^F6P^ for PFK-P and PFK-L, but markedly decreases *V*_*max*_^F6P^ for PFK-M. In contrast, ADP has little effect on affinity for ATP for PFK-P, but decreases affinity for PFK-L and PFK-M. In contrast, GDP has a negative effect, causing a loss of affinity for ATP in all isoforms. As with all the results discussed, comparison of these values to the published literature is challenging given the paucity of comparative studies, and differences in enzyme purification (tissue derived vs. recombinant) and assay conditions (e.g. ATP concentrations).

The allosteric sites for the three isoforms were compared in order to explore how sequence differences might explain the changes in AMP or ADP binding on enzyme activity (Supplementary Table S7). An overlay of the EcPFK structure (PDB 1PFK) defines two allosteric sites (A and B) in each human PFK (hPFK) chain (Figure 1). In EcPFK, ADP binds in a cleft between two chains, whereas in the PFK-P structure (PDB 4xz2) these sites bind phosphate, which overlays very closely with the β-phosphate of the ADP bound to EcPFK. The shape of this allosteric A-site pocket fits closely with the EcPFK pocket and there are no apparent steric restrictions to prevent fitting ADP for any of the three isoforms. In the A-site, there are 15 amino acids within 4 Å of the docked ADP, with the key phosphate-binding residues (R430, R434) conserved between all human isoforms. Comparing these 15 residues, the PFK-L and PFK-M isoforms are most similar, while the PFK-L and PFK-P isoforms are least similar (87% and 67% conservation of sequence identities, respectively). Site B comprises 17 residues within 4 Å of the docked ADP. There is an insertion of three residues in the three human isoforms (627KTTIQR632), which matches structurally with the *E. coli* sequence (213KKH215) and results in an additional bulge at the neck of the ADP binding pocket with Ile630 closing the entrance and potentially sterically hindering ADP binding. This tends to support the idea that the B-site may be more specific for smaller or more flexible effector molecules like PEP or citrate.

The pattern for activation summarised in Table 3 shows the PFK-L and PFK-P isoforms are both strongly activated by AMP and ADP, while PFK-M is slightly inhibited. AMP has a consistently greater effect than ADP. There are two substitutions in the B-site that differentiate PFK-M from the PFK-P and PFK-L isoforms (S571A and I630V) which may play a role in the difference in behaviour for PFK-M (Supplementary Table S7).

#### Allosteric modulation of PFK activity by citrate and PEP

The results presented here show considerable diversity of the effect of citrate on the different isoforms (Figure 4, Table 3, Supplementary Table S5). There are marked dose-response effects for PFK-M (IC_50_^citrate^ 982 ± 109 µM) and PFK-P (IC_50_^citrate^ 177 ± 132µM), but not PFK-L (no clear relationship). With respect to ATP, maximal activity decreases (especially for PFK-P), but ATP affinities increase. A clear picture of inhibition is demonstrated for F6P titrations, with decreasing maximal activities and increasing K_0.5_^F6P^ values. When specific activities are considered, the overall impression is that citrate acts as an activator with regards to ATP (PFK-P > PFK-L > PFK-M) at least at low (sub-physiological) ATP concentrations, but as an inhibitor with regards to F6P (PFK-P > PFK-M > PFK-L). Thus, citrate is not simply the pure inhibitor described in the literature.

PEP, previously described as a PFK inhibitor, also has mixed effects (Table 3, Supplementary Table S5). Although both PFK-M and PFK-L (but not PFK-P) show increased affinity for ATP in the presence of PEP, the concomitant decrease in V_max_^ATP^ cancels this out, with just PFK-P showing a marked inhibitory effect when specific activities are measured. Similarly, PFK-L and PFK-P (but not PFK-M) have decreased affinity for F6P in the presence of PEP, but changes in *V*_*max*_^F6P^ mean that overall effects are negligible, except for a modest activating effect on PFK-L.

Published studies have shown that mutations of PFK-M in the B-allosteric site (K567R, D601V and K627A, amino acid numbering conforming to Supplementary Table S7) preserved wild-type activity but were much less sensitive to the inhibitory effect of citrate [36]. Electron microscopy has been used to show that citrate promoted dissociation of wild-type PFK-P tetramers into dimers [8]. Another study showed that the cancer-associated mutation R48C reduced the inhibitory effect of citrate 10-fold. An earlier study also showed that another mutation of this phosphate-binding residue (R48L) abolished the inhibitory effects of citrate on rabbit PFK-M [37]. All four residues in these mutational studies are conserved across the M, L and P isoforms, strongly indicating that the B-site (Supplementary Table S7) is a conserved allosteric binding site for citrate. The observation that the R48L mutation had a slightly positive effect on the allosteric activity of ADP or AMP [37] further supports the idea that the B-site is regulated by citrate and possibly smaller metabolites while the A-site (Figure 1) is regulated by AMP and ADP [36].

The marked effects of citrate on PFK-P compared with the more modest effects on PFK-L and PFK-M (Figure 4, Table 3, Supplementary Table S5) may be explained by differences in amino acid sequences in the B-site. The 17 amino acids lining the citrate-binding B-site are conserved among the three isoforms by 76%–88%, with only one amino acid S84M (PFK-M) and S84N (PFK-L) uniquely non-conserved (Supplementary Table S7). Intra-cellular concentrations of F6P are generally far lower than ATP (e.g. 0.11 mM F6P in human muscle and 5.1 mM ATP in rat cardiomyocytes [34,38]), leading to the conclusion that citrate typically acts as an inhibitor under physiological conditions.

#### The role of tetramer thermal stability and PFK enzyme activity

The enzymatic activity of PFK-M declined rapidly in a concentration and time-dependent fashion (Supplementary Figure S1) in the absence of substrates (F6P or ATP) or reducing agent (TCEP). Storage at 4°C for 24 h led to a decrease in PFK-M activity to 52% of baseline when stored at a concentration of 0.32 mg/ml, and to 19% at 0.16 mg/ml, and 7% at 0.08 mg/ml. The decline in activity was inversely proportional to concentration. Addition of substrates (F6P or ATP) or reducing agents (TCEP or dithiothreitol, DTT) reduced the loss of activity. Simultaneous addition of substrate and a reducing agent to PFK-M concentrations above 0.1 mg/ml resulted in complete protection against inactivity, though concentrations below 0.1 mg/ml still lost activity over time (data not shown). Thermal denaturation assays demonstrated protein stability also correlated with protein concentration (data not shown). PFK-M tetramer was most stable in size-exclusion chromatography, but both PFK-L and PFK-P showed broad retention peaks with trailing edges, in keeping with on-column dissociation; PFK-P was especially susceptible to on-column dissociation, with markedly overlapping tetramer and dimer peaks (Supplementary Figure S2). Taken together, these results suggest a hierarchy of isoform stability with different rate constants for time-dependent dissociation: PFK-M being most stable, then PFK-L, and finally PFK-P.

These results showing the clear correlation between enzyme activity and tetramer stability are consistent with previously published data that used light scattering to measure the dimer-tetramer equilibrium for PFK-M [5]. Electron microscopy has also been used to directly visualise the dimer and tetramer populations of PFK-P, with a mutation (F649L) that weakens Interface 2 and may result in an increased proportion of dimers over tetramers (Supplementary Table S4) [8]. The mechanism by which allosteric modulators affect PFK activity remains uncertain. Previous studies describe how various modulators affect activity including activation by F26BP, ADP, and phosphate, and inhibition by high ATP concentrations and citrate [5,8,39]. The precise mechanism by which this occurs is unknown, and it remains up for debate; whether it is due to direct stabilisation/destabilisation of the tetramer/dimer/monomer states; whether it is due to changes in the conformational equilibria of the tetramer between R-state and T-state; or whether it is a combination of the two mechanisms.

In the comparative study of the three paralogous PFKs presented here, we show that PFK-P and PFK-L are highly sensitive to AMP and ADP activation, with PFK-M being much less affected. Interestingly, the trypanosomatid PFKs show similar disparate behaviour that is explained by differences in thermal stability: *Leishmania infantum* PFK (LiPFK) has a remarkable response to AMP compared with the related *Trypanosoma brucei* and *Trypanosoma cruzi* PFKs [27]. In the case of LiPFK the AMP response was linked to a dramatic increase in protein stability (as measured by thermal stability), favouring the formation of active tetramers, and preventing dissociation into inactive dimers. This is analogous to the allosterically regulated PFK-P and PFK-L isoforms, which are regulated (stabilised) by AMP/ADP binding to the allosteric A-site in the N and C-domains (Figure 1, Supplementary Table S7, and Supplementary Table S8).

### Specialised biological roles of the M, L and P isoforms

#### Transcript profiles of PFK isoforms show differential transcription according to tissue type

**Figure 5.**
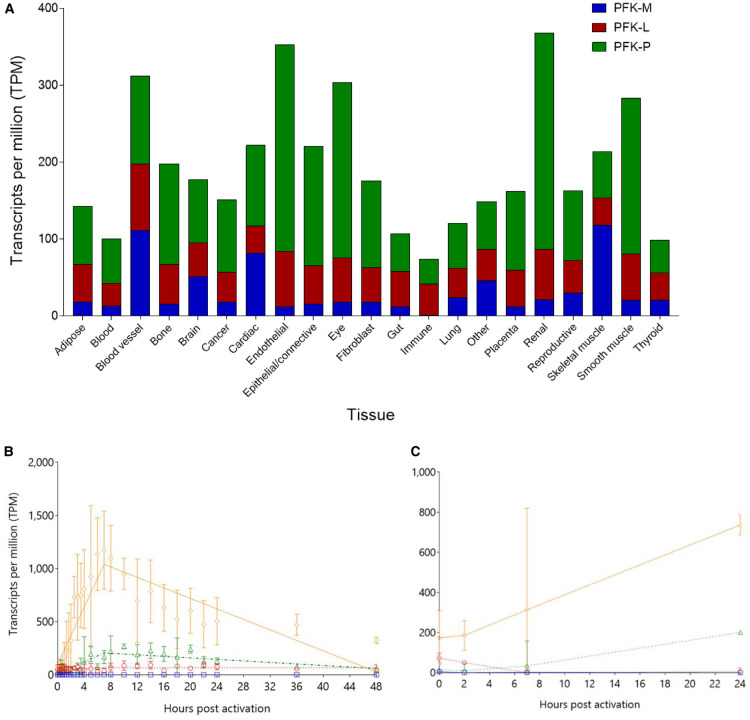
PFK isoforms show tissue specific dynamic differences in transcript levels. (**A**) Mean average TPM values for each group of FANTOM-5 samples were compared for each isoform. Certain tissues contain more PFK transcripts than other tissues e.g. renal, endothelium, and eye. Proportions of each isoform follow the general hierarchy of PFK-P > PFK-L > PFK-M, though with notable exceptions e.g. immune cells and skeletal muscle. Liver data are included under ‘Gut’ heading, and shows: 47.2% PFK-L; 47.1% PFK-P; 5.6% PFK-M. (**B**,**C**) PFK-P transcription overtakes PFK-L in monocyte-derived macrophages exposed to LPS (Panel **B**) and Influenza A (Panel **C**), with PFKFB3 correlating with PFK-P. PFK-M (blue squares 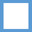 with solid lines); PFK-L (red circles 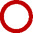 with dotted lines); PFK-P (green triangles 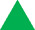 with dashed lines); PFKFB3 (orange diamonds 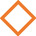 with dot-dashed lines). Lines are segmented lines of best fit, errors bars are standard deviations, *n* = 3 for LPS and *n* = 4 for influenza.

Transcript profiles of the three PFK isoforms differed greatly between the 21 tissue types included in the FANTOM-5 database (Figure 5A). Total transcript per million (TPM) scores demonstrate PFK-P transcripts to be the most abundant (106,128), followed by PFK-L (46,641) and PFK-M the least (23,454). This hierarchy (PFK-P > PFK-L > PFK-M) is found in most tissue types, except for brain and cardiac tissue (PFK-P > PFK-M > PFK-L), skeletal muscle (PFK-M > PFK-P > PFK-L), and immune cells (PFK-L > PFK-P > PFK-M). Liver cells show an equal predominance of PFK-L and PFK-P, with a much smaller contribution from PFK-M. The interpretation of these results is limited by the lack of full correlation between transcript profiles and protein levels, which are influenced by transcriptional efficiency, transcript stability, and protein turnover, amongst other factors, as shown by the cytoskeletal-mediated ubiquitin-dependent proteasomal degradation of PFK [40] and quantitative proteomic studies [41]. Correlation with semi-quantitative proteomic data was performed using the Human Tissue Atlas, which uses commercially available isoform-specific antibodies [42]. These data confirm that PFK-M and PFK-P are expressed at greater levels compared with PFK-L.

In immune cells, PFK-M transcripts are virtually absent, with the predominant isoform being PFK-L. Activation of monocyte-derived macrophages by lipopolysaccharide (LPS) or Influenza A results in dramatic changes in PFK isoform transcript levels (Figure 5B,C). PFK-P is up-regulated by LPS (10.3× baseline) and Influenza A (13.8×); PFKFB3 transcript levels closely mirror PFK-P. In contrast, PFK-L transcript levels increase modestly after LPS stimulation (1.9× baseline) and decrease to negligible levels after Influenza A infection. Active immune cells require large amounts of energy for the ATP-dependent process of cell membrane movement (phagocytosis) and for the NADPH-dependent respiratory burst and production of neutrophil extracellular traps, both dependent on glycolysis and glutaminolysis [43,44]. Thus, the switch from PFK-L at rest to PFK-P during activation is a strong clue to the differing functions of each isoform. The relative insensitivity of PFK-P to ATP inhibition would permit high enzymatic activity irrespective of cellular ATP concentrations, as opposed to PFK-L which is more responsive to ATP inhibition.

#### Correlations between transcript levels of metabolic enzymes show cancer-related changes

Transcript levels of PFK-L and PFK-P were positively correlated in non-cancer tissues (*r* = 0.68, *P* = 7.1 × 10^−4^), but there was no statistically significant relationship between either of these isoforms and PFK-M. This suggests that PFK-P and PFK-L are not ‘rivals’ but instead work together, having different metabolic roles within cells. This links to the kinetic and regulatory properties of each isoform: since PFK-P is responsive in low ATP conditions, and PFK-L in low F6P conditions, expressing both simultaneously will expand the metabolic capacity for the F6P to F16BP reaction under varying conditions. PFK-M alone would have the same capacity but lacks the regulatory activity of the other two isoforms, particularly with regards to activation by F26BP. In cancer tissues, the correlation between PFK-L and PFK-P becomes non-significant (*r* = 0.14, *P* = 0.39), suggesting that cancer tissues have a more binary approach to PFK expression, prioritising one isoform over another. Full correlation coefficients are shown in Supplementary Tables S9A,B. An alternate explanation for the positive correlation between PFK-L and PFK-P transcript levels is that either or both isoforms have ‘moonlighting’ functions — secondary cellular roles distinct from that in glycolysis. Moonlighting functions are found in many glycolytic enzymes [45,46], including PFK-P, which also acts as a co-transcriptional regulator of YAP/Taz and an activator of PI3K [47,48]. Increased transcript levels may therefore relate to non-glycolytic functions.

In non-cancerous tissues, transcript levels of PFK-P correlate highly (0.67) with the pyruvate kinase (PYK) isoenzymes M1 and M2 (PKM1/2). In cancerous tissues this is the only strong correlation to be preserved among the glycolytic enzymes. This analysis does not distinguish between the M1 and M2 PYK isoforms, although previous studies have shown that it is the allosterically regulated M2 splice variant form that is prevalent in cancer tissues [20]. Our work highlights several interesting similarities between PYK and PFK — long regarded as the key regulators of the glycolytic pathway. Of the two forms of PYK-M, M1 is constitutively active — analogous to PFK-M — whereas M2 is allosterically regulated (by F16BP) through dissociation of active tetramers into inactive dimers/monomers [49], analogous to the behaviour of PFK-P (and PFK-L), which are allosterically regulated by F26BP preventing dissociation of active tetramers into inactive dimers/monomers. Interestingly it is the highly allosterically regulated forms of both PFK (PFK-P) and PYK (PKM2) that are up-regulated in cancer cells, presumably allowing glycolysis to work efficiently when nutrients are available but also shut down rapidly in response to changes in metabolite concentration.

The relationship between PFKFB3, responsible for synthesis of F26BP, the most potent enhancer of PFK activity, and PFK-P differs in non-cancer and cancer tissues. In non-cancerous tissues there is a negative correlation between transcripts levels of these enzymes, suggesting a regulatory mechanism that limits overall PFK activity (high expression of PFK is counteracted by lower F26BP levels). In cancer tissues there is a loss of this potential feedback mechanism: transcript levels of PFK and PFKFB3 are positively correlated. This highlights the physiological relevance of the highly activating effect of F26BP demonstrated in the kinetic experiments above and may provide an explanation for why PFK-P — the isoform most strongly activated by F26BP — is most up-regulated in cancers. LDH-A, another cancer-associated enzyme, also positively correlates with PFK-P [50,51].

The position of glucose-6-phosphate dehydrogenase (G6PDH) as the entry-point to the anabolic pentose-phosphate pathway (PPP) means that transcript levels of this enzyme could be expected to negatively correlate with downstream glycolytic enzyme transcript levels. However, the correlation data indicate the reverse: G6PDH is positively correlated with most glycolytic enzymes, including PFK-L and PFK-P (*r* = 0.64, *P* = 2 × 10^−3^; *r* = 0.47, *P* = 0.03, respectively). There is no correlation between G6PDH and PFKFB3 (which increases glycolytic activity through activation of PFK), suggesting a lack of antagonism between the PPP and glycolytic pathways. This supports the concept that there is not a binary choice between glycolysis and the PPP: what occurs may be an up-regulation of both pathways at the same time. Thus, the cell may literally both have its cake (making new cellular building blocks) and eat it (making energy available), an idea currently in vogue [52]. These correlations are not found in cancer samples, suggesting prioritisation of single pathways in cancer tissues.

## Conclusions

In this paper we present the first systematic comparison of recombinant human PFK isoforms. Intrinsic differences in substrate affinities for each isoform were demonstrated. PFK-M is the most active enzyme but is much less versatile than the other isoforms, being unable to respond to allosteric modulation to the degree shown by PFK-L and PFK-P, especially with regard to F26BP activation and ATP inhibition. Comparison of the amino acid sequences of the three isoforms using published X-ray structures supports the idea that variations in isoform activity and regulation are linked to tetrameric stability at the key dimer-dimer interface (Interface 2 [Figure 1]), which controls the active tetramer to inactive dimer equilibrium.

The biological rationale for these structural and kinetic differences evolving and persisting was explored by investigating tissue-specific transcript levels of each isoform. PFK-M, which is most active but least regulated, is the predominant isoform in skeletal muscle, where there is a constant need for ATP production. Brain and cardiac muscle, which also have high baseline ATP requirements, also express high levels of PFK-M, whereas PFK-P is the dominant isoform in most tissues, highlighting the importance of activity modulation by allosteric regulators.

Extending these comparisons to include cancer-derived samples reveals further insights into the role of PFK. Transcript levels of PFK-P strongly correlate with PYK isoforms in both non-cancerous and cancer samples, with intriguing similarities between PFK-M and PKM1 (highly active, poorly regulated), and also between PFK-P and PKM2 (lower activity, greater regulation). It is the highly regulated low activity isoforms — PFK-P and PKM2 — that are associated with cancer samples, indicating that for some cancers glycolytic regulation may be of greater importance than activity.

## Footnotes

1 https://www.ebi.ac.uk/msd-srv/prot_int/pistart.html

## Open Access

Open access for this article was enabled by the participation of University of Edinburgh in an all-inclusive Read & Publish pilot with Portland Press and the Biochemical Society under a transformative agreement with JISC.

## Abbreviations

EcPFK: *Escherichia coli* PFK
F16BP: fructose 1,6-bisphosphate
F26BP: fructose 2,6-bisphosphate
G6PDH: glucose-6-phosphate dehydrogenase
LPS: lipopolysaccharide
LiPFK: *Leishmania infantum* PFK
PFK: 6-Phosphofructokinase-1-kinase
PKM2: pyruvate kinase M2
PPP: pentose-phosphate pathway
PYK: pyruvate kinase
TCEP: Tris(2-carboxyethyl)phosphine hydrochloride
TPM: Transcripts per million
TbPFK: *Trypanosoma brucei* PFK
