# Supplementary material for: Biochemical and transcript level differences between the three human phosphofructokinases show optimisation of each isoform for specific metabolic niches
Source: Biochem J. 2020 Nov 27;477(22):4425–41. doi: 10.1042/BCJ20200656 (PMC7702303; doi:10.1042/BCJ20200656)
Supplement: Supplementary Figures S1-S2 and Supplementary Tables S1-S10 [file BCJ-477-4425-s1.pdf]

Supplementary data to

SUPPLEMENTARY FIGURE 1: PFK-M activity decreases in a time and concentration dependent manner

PFK-M was stored at varying concentrations for 24 hours at 4°C. Activity was assessed using the standard kinetic assay described in the Methods section at 0 hours and 24 hours, using identical conditions. Results were normalized to 0 hours for each concentration. PFK-M activity declined over time, with 52% of original activity remaining after 24 hours storage at 4•C and 0.32mg/ml. Furthermore, this time dependent inactivation is also concentration dependent, since the effect is more marked at lower concentrations: activity is 19% at 0.16mg/ml and 7% at 0.08mg/ml. The decrease in activity is linear for the concentrations tested, fitting a model of $y = 1.895x - 0.1$, where y is percentage of original activity and x is concentration in mg/ml.

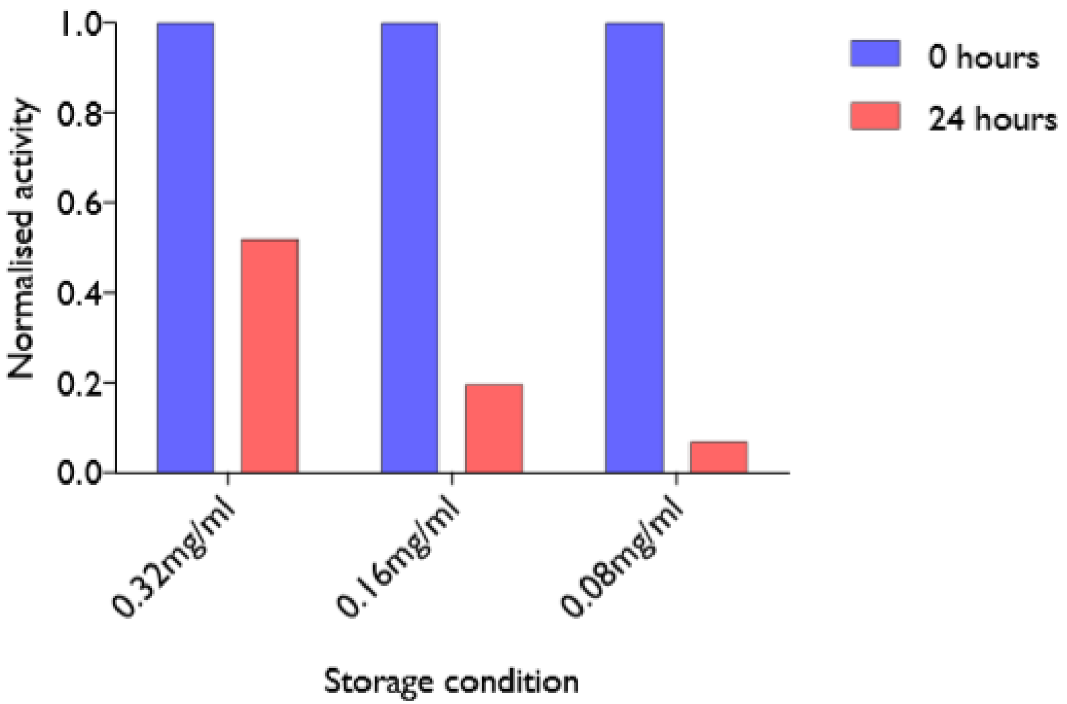
Normalised activity
1.0
0.8
0.6
0.4
0.2
0.0
0.32mg/ml
0.16mg/ml
0.08mg/ml
Storage condition
0 hours
24 hours

SUPPLEMENTARY FIGURE 2: Human PFK isoforms show differing stability using semi-analytical gel filtration

Semi-analytical gel filtration was used to characterize each isoform according to size (larger species elute before smaller species). Comparison of the three human isoforms show different tetramer-dimer equilibria for each paralogue. PFK-P is shown to be less stable than PFK-M or PFK-L. Panel A shows PFK-M; Panel B shows PFK-L; Panel C shows PFK-P. Blue is absorbance at 280nm in milli-Absorbance units (mAu), red is absorbance at 260nm in mAu, brown is conductivity [scale not shown], x-axis is elution volume in ml.

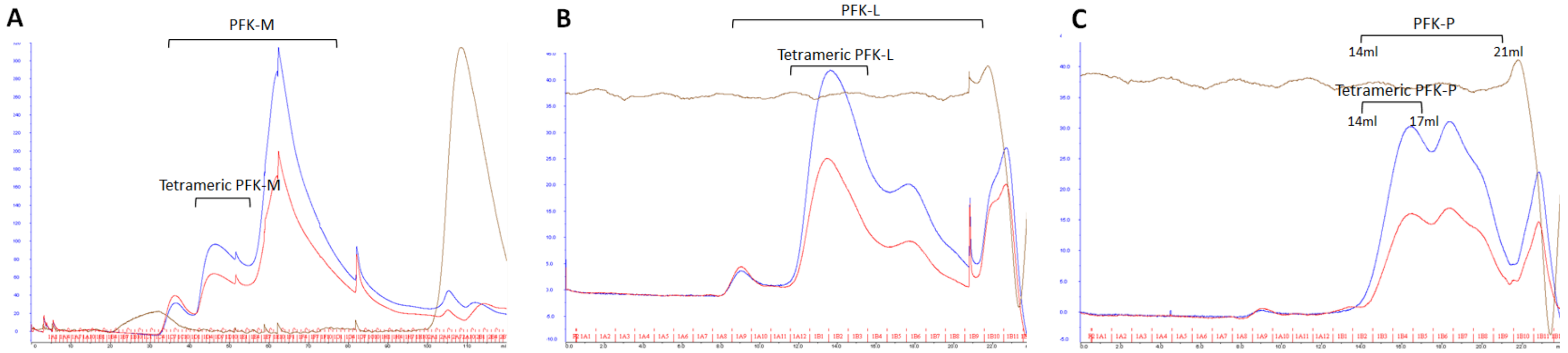

SUPPLEMENTARY TABLE 1: Summary of natural and artificial modulators of PFK activity in the published literature

PFK is allosterically regulated by many compounds ("+" indicates activator; "-" indicates inhibitor), with selected references.

Effector	Effect	IC_{50} or AC_{50}	Reference
Ions			
**NH_4^+**	+	0.4mM (PFKP)	Sanchez-Martinez et al., 2000
**PO_4^{3-}**	+	0.3mM (PFKP)	Sanchez-Martinez et al., 2000
**K^+**	+	N/A	Moreno-Sanchez et al., 2012
Metabolites			
**Acyl-CoA**	-	2-7μM (PFKM)	Jenkins et al., 2011
**ADP**	+	N/A	Passoneau and Lowry 1962
**AMP**	+	8μM (PFKP)	Sanchez-Martinez et al., 2000
		10μM (PFKM)	Foe and Kemp, 1985
		10μM (PFKL)	Foe and Kemp, 1985
		75μM (PFKP)	Foe and Kemp, 1985
		0.86/5.8/1.55mM*	Moreno-Sanchez et al., 2012
**ATP**	-	N/A	Sanchez-Martinez et al., 2000
		1.1/2.9/1.75mM*	Moreno-Sanchez et al., 2012
**Ascorbate**	-	N/A	Russell et al., 2009
**cAMP**	+	N/A	Pinilla and Luque, 1981
**cGMP**	-	N/A	Pinilla and Luque, 1981
**Citrate**	-	400μM (PFKP)	Sanchez-Martinez et al., 2000
		100μM (PFKM)	Foe and Kemp, 1985
		>2000μM (PFKL)	Foe and Kemp, 1985

		750μM (PFKP)<br>130μM (PFKM)<br>180μM (PFKL)<br>80μM (PFKP)<br>5.9/0.49/6.7mM*	Foe and Kemp, 1985<br>Vora 1985<br>Vora 1985<br>Vora 1985<br>Moreno-Sanchez et al., 2012
**F16BP**	+	Kact 1.5μM (PFKM)<br>40μM (PFKL)	Sanchez-Martinez et al., 2000<br>Van Schaftingen et al 1981
**F26BP**	+	Kact 2.2μM (PFKP)<br>0.04μM (PFKL)<br>0.05μM (PFKM)<br>0.05μM (PFKL)<br>4.5μM (PFKP)<br>10 (PFKM)<br>100 (PFKL)<br>100 (PFKP)<br>0.53/ 4.6/0.58*	Sanchez-Martinez et al., 2000<br>Van Schaftingen et al 1981<br>Foe and Kemp, 1985<br>Foe and Kemp, 1985<br>Foe and Kemp, 1985<br>Vora 1985<br>Vora 1985<br>Vora 1985<br>Moreno-Sanchez et al., 2012
**G16BP**	+	N/A<br>100uM (PFKL)	Meienhofer et al., 1980<br>Van Schaftingen et al 1981
**Lactate**	-	4.6mM*	Costa Leite et al., 2007
**PEP**	-	Ki 2.78mM (PFKP)	Sanchez-Martinez et al., 2000
Proteins			
**Actin**	+	N/A	Liou and Anderson, 1980
**Aldolase**	+	N/A	Marcondes et al., 2011
**Band 3**	-	N/A	Messana et al., 1996

**Calmodulin**	+ & -	N/A	Marinho-Carvalho et al., 2006
**Parathymosin-α**	-	N/A	Brand and Soling, 1986
**Tubulin**	-	N/A	Ovhdi et al., 1996
Drugs			
**Aspirin**	-	2.3mM*	Spitz et al., 2009
**Bupivacaine**	-	N/A	Schwartz and Beitner 2000
**Clotrimazole**	-	N/A	Guimarães et al., 2011
**Lidocaine**	-	N/A	Schwartz and Beitner 2000
**Paclitaxel**	-	N/A	Glass-Marmor and Beitner, 1999
**Vinblastine**	-	N/A	Vertessy et al, 1998

* indicates isoform not specified or not identified

SUPPLEMENTARY TABLE 2: Comparison of physiological and tested effector concentrations

The concentrations selected was based on two factors: the normal physiological concentrations of the effector, and the chance of seeing an effect on activity at a given concentration. There were often discrepancies between these two criteria; a compromise was made in these cases, with preference given to concentrations which were likely to demonstrate effects over and above the signal-to-noise threshold for this assay. For some effectors there is significant uncertainty – and sometimes even controversy – about physiological concentrations e.g. citrate.

Metabolite	***Physiological intracellular concentrations***	***Tested concentrations***	***References for physiological concentrations***
**AMP**	82µM	1200µM	(1)
**ADP**	138-1500µM	600µM	(2)
**GDP**	36µM	1100µM	(1)
**F26BP**	2.5-5µM	10µM	(3)
**Citrate**	80-500µM	600µM	(4,5)
**PEP**	23µM	1100µM	(2)
**F6P**	0.11mM<br>(much higher in liver)	N/A	(2,6)
**ATP**	5.1mM in rat cardiomyocytes (human average 2.1mM)	N/A	(1)

SUPPLEMENTARY TABLE 3: Comparison of Interface 1 (D-C) amino acid sequence between PFK-M, PFK-L and PFK-P

Interface 1 (D-C interface) amino acid sequence comparison between PFK-M, PFK-L, and PFK-P, incorporating all residues contributing more than 4 Å^2 buried surface area (BSA) to the interface based on the X-ray structure of PFK-P (PDB code 4xz2). Column 1 has the name of the residue in the X-ray structure. Yellow highlights non-conserved amino acids across the three isoforms. Blue indicates interface residues that also interact with FBP. HB indicates the residue forms a hydrogen bond across the interface.

	PFK-M	PFK-L	PFK-P	BSA (Å^2) (D-C)
D:GLY 33	G	G	G	6
D:GLY 34	G	G	G	4
D:ASP 35	D	D	D	37
D:GLU 62	E	E	E	46 HB
D:VAL 88	L	L	V	13
D:GLY 89	G	G	G	15
D:GLY 90	G	G	G	13
D:THR 91	T	T	T	21 HB
D:ILE 92	V	I	I	20
D:GLY 94	G	G	G	15
D:SER 95	S	S	S	31 HB
D:ALA 96	A	A	A	6
D:VAL 197	V	I	V	21
D:ALA 200	A	A	A	55 HB
D:ILE 201	I	I	I	26
D:THR 203	T	T	T	42 HB
D:THR 204	T	T	T	62 HB
D:SER 207	S	S	S	29 HB
D:HIS 208	H	H	H	61 HB
D:ARG 210	R	R	R	17
D:PHE 212	F	F	F	16
D:ARG 265	R	R	R	26 HB
D:ARG 301	R	R	R	30
D:THR 303	T	T	T	33
D:ILE 304	V	V	I	24
D:LEU 305	L	L	L	7

D:GLY 306	G	G	G	4
D:HIS 307	H	H	H	99
D:VAL 308	V	V	V	74
D:ARG 310	R	R	R	27 HB
D:GLY 311	G	G	G	25 HB
D:GLY 312	G	G	G	34
D:PRO 421	P	P	P	48
D:ASP 448	E	E	D	22 HB
D:GLY 473	G	G	G	19
D:GLY 474	G	G	G	31
D:SER 475	S	S	S	27
D:ILE 476	K	M	I	11
D:LEU 477	L	L	L	4 HB
D:GLY 478	G	G	G	16
D:THR 479	T	T	T	20 HB
D:LYS 480	K	K	K	34 HB
D:THR 558	T	A	T	18
D:THR 562	T	S	T	22
D:ARG 565	R	R	R	107 HB
D:ILE 566	I	I	I	15
D:GLN 568	Q	Q	Q	84 HB
D:SER 569	S	S	S	57 HB
D:SER 571	A	S	S	4
D:GLY 572	G	G	G	65 HB
D:THR 573	T	T	T	55 HB
D:LYS 574	K	K	K	75 HB
D:ARG 575	R	R	R	8
D:ARG 576	R	R	R	10
D:PHE 578	F	F	F	14
D:ARG 665	R	R	R	21
D:ASN 667	N	N	N	49 HB
D:VAL 668	V	V	V	25
D:LEU 669	L	L	L	5
D:GLY 670	G	G	G	11
D:HIS 671	H	H	H	108 HB

D:MET 672	M	L	M	113 HB
D:GLN 674	Q	Q	Q	17 HB
D:GLY 675	G	G	G	32
D:GLY 676	G	G	G	47
D:ALA 677	S	A	A	16

The C-D interface comprises 75 residues from chain D and 71 from chain C. The BSA D = 2164 $Å^2$ and for chain C = 2196 $Å^2$ with 40 h-bonds including 9 salt bridge interactions. The A-B interface comprises 73 residues from chain B and 67 from chain A. The BSA B = 2113 $Å^2$ and for chain A = 2158 $Å^2$ with 34 h-bonds including 7 salt bridge interactions. There are 66 residues that contribute to this interface with a BSA of at least 4 $Å^2$: PFK-P differs from PFK-M by 6/66 residues; PFK-P differs from PFK-L by 7/66 residues; PFK-L differs from PFK-M by 8/66 residues. Between P and M most of the changes (I90V, V91I, D448E) are conservative. A677S, S571A, I476K are the only three difference between isoforms P and M that are not conservative.

SUPPLEMENTARY TABLE 4: Interface 2 (D-A and B-C) amino acid sequence comparison

Interface 2 amino acid sequence comparison. The output from PISA (http://www.ebi.ac.uk/pdbe/prot_int/pistart.html) using the coordinates from PFK-P (PDB code 4xz2) was used to determine BSA values for each of the 19 residues associated with the D-A and B-C interfaces. All residues contributing a BSA value greater than 0.5 Å^2 were included in the analysis. Yellow highlighting indicates non-conserved amino acid. HB indicates the residues forms a hydrogen bond across the interface.

	PFK-M	PFK-L	PFK-P	BSA (Å^2) (D-A) & (B-C)
D:PHE 610	F	F	F	5.9
D:ASP 611	T	N	D	12.8
D:ILE 612	I	I	I	79.3 HB
D:ARG 613	R	H	R	47.8 HB
D:GLN 616	Q	K	Q	64.5 HB
D:GLU 620	E	E	E	10.3 HB
D:SER 642	N	H	S	8.6
D:GLU 643	E	D	E	4.1
D:ASN 644	N	Y	N	99.9
D:TYR 645	Y	Y	Y	44.9 HB
D:THR 646	T	T	T	13.9
D:PHE 649	F	F	F	65.5
D:TYR 651	F	Y	Y	6.4
D:GLN 652	N	N	Q	80.5
D:LEU 653	L	L	L	34.4
D:SER 655	S	S	S	12.8 HB
D:GLU 656	E	S	E	63.8
D:GLU 657	E	E	E	63.8 HB
D:LYS 659	K	K	K	16.8

There are 11/19 (58%) residues conserved between P and L, and 15/19 (79%) residues conserved between P and M at this interface (12/19 conserved between M and L). The A-D interface comprises 19 residues from chain A and the same 19 residues from chain D. There are 8 hydrogen bonds incorporating 4 salt bridges. The BSAs of chain D and chain A are 736 Å^2 and 708 Å^2, respectively. (The interface area is given as 721 Å^2, the average of the two). The B-C interface comprises 19 residues from chain B and the same residues from chain C. There are 7 hydrogen bonds incorporating 4 salt bridges.

SUPPLEMENTARY TABLE 5: Summary of kinetic parameters for each human PFK isoform with and without various natural modulators of PFK activity

Effect of modulators of PFK activity. For ATP titrations: F6P 4mM; for F6P titrations: ATP 0.5mM. N=3, values are mean averages (with standard deviations in parentheses). Respective control experiments (without modulators) were performed simultaneously alongside modulator experiments to minimise the confounding effects of time dependent dissociation on comparisons.

	PFK-M	***PFK-L***	***PFK-P***
V_{max}^{ATP} (μmoles/min/mg)	37.55 (1.42)	39.26 (1.51)	30.74 (2.92)
+ AMP 1200μM (μmoles/min/mg)	37.45 (1.41)	44.45 (1.74)	28.38 (1.07)
+ ADP 600μM (μmoles/min/mg)	34.91 (1.76)	49.38 (2.48)	37.99 (1.74)
+ F26BP 10μM (μmoles/min/mg)	38.80 (0.75)	38.57 (1.23)	31.26 (1.96)
+ citrate 600μM (μmoles/min/mg)	35.65 (1.36)	36.14 (1.12)	10.18 (0.89)
V_{max}^{F6P} (μmoles/min/mg)	6.33 (0.07)	3.60 (0.08)	1.65 (0.17)
+ AMP 1200μM (μmoles/min/mg)	2.85 (0.04)	5.68 (0.06)	2.13 (0.06)
+ ADP 600μM (μmoles/min/mg)	3.46 (0.09)	5.65 (0.07)	2.51 (0.11)
+ F26BP 10μM (μmoles/min/mg)	5.81 (0.09)	5.07 (0.41)	2.45 (0.18)
+ citrate 600μM (μmoles/min/mg)	6.00 (0.11)	6.52 (0.71)	0.43 (0.12)
**$K_{0.5}^{ATP}$ *(μM)***	148.0 (16.7)	151.2 (16.9)	326.9 (99.7)
+ AMP 1200μM (μM)	82.4 (4.2)	156.9 (17.6)	94.4 (10.8)
+ ADP 600μM (μM)	289.8 (37.7)	362.0 (46.5)	290.5 (36.0)
+ F26BP 10μM (μM)	165.2 (9.2)	139.4 (12.3)	176.8 (33.8)
+ citrate 600μM (μM)	133.8 (14.5)	108.4 (9.5)	46.0 (14.4)
**$K_{0.5}^{F6P}$ *(μM)***	136.7 (4.2)	1432 (34)	1238 (179)
+ AMP 1200μM (μM)	82.39 (4.20)	260.6 (8.2)	607.0 (31.0)

+ ADP 600μM (μM)	86.0 (6.8)	872.9 (14.9)	821.7 (53.4)
+ F26BP 10μM (μM)	109.6 (5.3)	698 (105)	2143 (132)
+ citrate 600μM (μM)	202 (8.1)	2471 (244)	1494 (627)

	PFK-M	***PFK-L***	***PFK-P***
V_{max}^{ATP} ***(μmoles/min/mg)***	39.32 (0.84)	39.36 (1.80)	23.65 (2.40)
+ PEP 1100μM (μmoles/min/mg)	31.93 (1.10)	34.91 (1.59)	23.07 (2.40)
+ GDP 1100μM (μmoles/min/mg)	76.26 (34.54)	44.20 (2.84)	31.48 (4.42)
V_{max}^{F6P} ***(μmoles/min/mg)***	4.80 (0.07)	3.75 (0.13)	0.87 (0.04)
+ PEP 1100μM (μmoles/min/mg)	5.17 (0.08)	5.36 (0.49)	0.81 (0.04)
$K_{0.5}^{ATP}$ ***(μM)***	155.82 (9.83)	169.0 (21.5)	217.9 (74.5)
+ PEP 1100μM (μM)	142.3 (14.3)	148.7 (18.6)	352.3 (133.2)
+ GDP 1100μM (μM)	5820 (6634)	404.4 (65.6)	924.6 (367.7)
$K_{0.5}^{F6P}$ ***(μM)***	164.3 (6.3)	1282 (53)	1607 (90)
+ PEP 1100μM (μM)	191.3 (8.3)	952.8 (137.7)	1378 (96)

SUPPLEMENTARY TABLE 6: Comparison of F26BP binding sites between PFK-M, PFK-L, and PFK-P.

Output from PISA (http://www.ebi.ac.uk/pdbe/prot_int/pistart.html) using the coordinates from PFK-P (PDB code 4xz2) was used to determine Buried Surface Area (BSA) values for each of the residue close to F26BP (results are very similar for all chains in the tetramer and only one set is shown). All residues contributing a BSA value greater than 0.5 Å^2 were included in the analysis. 5/19 residues are involved in both binding F26BP and Interface 1 (coloured blue). No residues that bind F26BP are involved in Interface 2. B indicates Hydrogen Bond.

	PFK-M	PFK-L	PFK-P	BSA (Å^2) (D-C)
B:ALA 420	A	A	A	
B:PRO 421	P	P	P	
B:ARG 481	R	R	R	HB
B:GLY 508	G	G	G	
B:PHE 509	F	F	F	
B:GLU 510	E	E	E	
B:THR 538	T	T	T	HB
B:VAL 539	V	I	V	
B:SER 540	S	S	S	HB
B:ASN 542	N	N	N	
B:MET 583	M	V	M	
B:GLY 584	A	T	G	
B:GLY 585	G	G	G	
B:GLU 639	E	E	E	
B:HIS 671	H	H	H	
B:GLN 674	Q	Q	Q	HB
B:ARG 744	R	R	R	
A R576	R	R	R	
A R665	R	R	R	

SUPPLEMENTARY TABLE 7

Supplementary Table 4 shows the residues identified within 4Å from ADP in the X-ray structure of EcPFK (PDB code 1PFK). Structural and sequence comparisons were used to identify the corresponding residues in the two pockets (labelled Site A and Site B in Figure 1C) in PFK using the numbering in PFK-P (PDB 4xz2). Shaded boxes indicate which residues at the ADP binding site in EcPFK are type-conserved among the isoforms. Residues shown in red are amino acids identified by PISA (http://www.ebi.ac.uk/pdbe/prot_int/pistart.html) to be involved in binding phosphate.

	Site A					Site B			
EcPFK<br>PDB<br>(1PFK)	hPFKP<br>PDB<br>(4xz2)	PFK-M	PFK-L	PFK-P	EcPFK<br>PDB<br>(1PFK)	hPFKP<br>PDB<br>(4xz2)	PFK-M	PFK-L	PFK-P
R21	R430	R	R	R	R21	R44	R	R	R
R25	R434	R	R	R	R25	R48	R	R	R
R54	W463	W	W	W	R54	W79	W	W	W
Y55	T464	S	H	T	Y55	E80	E	L	E
S58	G467	G	A	G	S58	S83	S	S	S
D59	G468	G	G	G	D59	S84	M	N	S
R154	M202	T	T	M	R154	K567	K	K	K
S158	Q206	Q	Q	Q	S158	S571	A	S	S
G185	G233	G	G	G	G185	G599	G	G	G
E187	D235	D	D	D	E187	D601	D	D	D
K211	R262	R	R	R	K211	K625	K	K	K
G212	K263	G	G	K	G212	M626	M	M	M
K213	K264	S	S	K	K213	K627	K	K	K
K214	R265	R	R	R	K214	T628	T	T	T
						T629	T	D	T
						I630	V	I	I
H215	L266	L	L	L	H215	R632	R	R	R
Conservation<br>With EcPFK		6/15<br>40%	6/15<br>40%	7/15<br>47%			9/15<br>60%	10/15<br>68%	10/15<br>68%
Conservation<br>with P		12/15<br>80%	10/15<br>67%				14/17<br>82%	15/17<br>88%	
Conservation<br>With M			13/15<br>87%	12/15<br>80%				13/17<br>76%	14/17<br>82%

SUPPLEMENTARY TABLE 8A: N-terminal alignment of human PFK-P with EcPFK

Alignment of EcPFK (PDB 1PFK) with N-terminal domains of human PFK-M, PFK-L, and PFK-P isoforms. Residues in human PFK-P (PDB 4XZ2) involved in ligand binding of tetramer interfaces were identified with PISA (http://www.ebi.ac.uk/pdbe/prot_int/pistart.html). Alignments performed with Clustal Omega (https://www.ebi.ac.uk/Tools/msa/clustalo/)

--- Residues involved in Interface 1 and identified in Supplementary Table 3

--- Residues involved in Interface 2 and identified in Supplementary Table 4

--- Active site residues partially buried by ADP or F6P

--- F26BP binding site residues identified in Supplementary Table 6

--- Effector site B (Figure 1 and Supplementary Table 7) highlighting residues from EcPFK (PDB 1pfk) within 4 Å of effector ADP.

--- Effector site A (Figure 1 and Supplementary Table 7) highlighting residues from EcPFK (PDB 1pfk) within 4 Å of effector ADP

```
1PFK_A|PDBID|CHAIN|SEQUENCE   M----------------------IKKIGVLTSGGDAPGMNAAIRGVVRSA 27
sp|P08237.2|PFKAM_HUMAN       MTHEEHHAKT----------LGIGKAIAVLTSGGDAQGMNAAVRAVVRVG
sp|P17858.6|PFKAL_HUMAN       MAAVDLEKLRA---------SGAGKAIGVLTSGGDAQGMNAAVRAVTRMG
sp|Q01813.2|PFKAP_HUMAN       MDADDSRAPKGSLRKFLEHLSGAGKAIGVLTSGGDAQGMNAAVRAVVRMG 50
                              *                       * *.******** *****:*.*.* .

1PFK_A|PDBID|CHAIN|SEQUENCE   LTEGLEVMGIYDGYLGLYE--DRMVQLDRYSVSDMINRGGTFLGSARFPE 75
sp|P08237.2|PFKAM_HUMAN       IFTGARVFFVHEGYQGLVDGGDHIKEATWESVSMMLQLGGTVIGSARCKD
sp|P17858.6|PFKAL_HUMAN       IYVGAKVFLIYEGYEGLVEGGENIKQANWLSVSNIIQLGGTIIGSARCKA
sp|Q01813.2|PFKAP_HUMAN       IYVGAKVYFIYEGYQGMVDGGSNIAEADWESVSSILQVGGTIIGSARCQA 100
                              :  * .*  :::** *: :  ..: :    *** :::-***.:****

1PFK_A|PDBID|CHAIN|SEQUENCE   FRDENIRAVAIENLKKRGIDALVVIGGDGSYMGAMRLTE-----------
sp|P08237.2|PFKAM_HUMAN       FREREGRLRAAYNLVKRGITNLCVIGGDGSLTGADTFRSEWSDLLSDLQK
sp|P17858.6|PFKAL_HUMAN       FTTREGRRAAAYNLVQHGITNLCVIGGDGSLTGANIFRSEWGSLLEELVA
sp|Q01813.2|PFKAP_HUMAN       FRTREGRLKAACNLLQRGITNLCVIGGDGSLTGANLFRKEWSGLLEELAR 150
                              *  .: *  *  **  ::**  * *******  **  : .

1PFK_A|PDBID|CHAIN|SEQUENCE   ------------MGFPCIGLPGTIDNDIKGTDYTIGFFTALSTVVEAIDR 152
sp|P08237.2|PFKAM_HUMAN       AGKITDEEATKSSYLNIVGLVGSIDNDFCGTDMTIGTDSALHRIMEIVDA
sp|P17858.6|PFKAL_HUMAN       EGKISETTARTYSHLNIAGLVGSIDNDFCGTDMTIGTDSALHRIMEVIDA
sp|Q01813.2|PFKAP_HUMAN       NGQIDKEAVQKYAYLNVVGMVGSIDNDFCGTDMTIGTDSALHRIIEVVDA 200
```

```
                                           :   *: *:****: *** ***  :**  ::*-:*-

1PFK_A|PDBID|CHAIN|SEQUENCE  LRDTSSSHQRISVVEVMGRYCGDLTLAAAIAGGCEFVVVPEVEF---SRE 199
sp|P08237.2|PFKAM_HUMAN      ITTTAQSHQRTFVLEVMGRHCGYLALVTSLSCGADWVFIPECPPDDDWEE
sp|P17858.6|PFKAL_HUMAN      ITTTAQSHQRTFVLEVMGRHCGYLALVSALASGADWLFIPEAPPEDGWEN
sp|Q01813.2|PFKAP_HUMAN      IMTTAQSHQRTFVLEVMGRHCGYLALVSALACGADWVFLPESPPEEGWEE 250
                             : -*:.**** -*:*****:** *:*.:::: *.:::.:**       .:

1PFK_A|PDBID|CHAIN|SEQUENCE  DLVNEIKAGIAKGKKHAIVAITEHMCDVD-------ELAHFIEKETGRET 242
sp|P08237.2|PFKAM_HUMAN      HLCRRLSETRTRGSRLNIIIVAEGAIDKNGKPITSEDIKNLVVKRLGYDT
sp|P17858.6|PFKAL_HUMAN      FMCERLGETRSRGSRLNIIIIAEGAIDRNGKPISSSYVKDLVVQRLGFDT
sp|Q01813.2|PFKAP_HUMAN      QMCVKLSENRARKKRLNIIIVAEGAIDTQNKPITSEKIKELVVTQLGYDT 300
                              :  .:    :: .:  *: ::*   * :        : .::  . * :*

1PFK_A|PDBID|CHAIN|SEQUENCE  RATVLGHIQRGGSPVPYDRILASRMGAYAIDLLLAGYGG--RC-VGIQNE 189
sp|P08237.2|PFKAM_HUMAN      RVTVLGHVQRGGTPSAFDRILGSRMGVEAVMALLEGTPDTPACVVSLSGN
sp|P17858.6|PFKAL_HUMAN      RVTVLGHVQRGGTPSAFDRILSSKMGMEAVMALLEATPDTPACVVTLSGN
sp|Q01813.2|PFKAP_HUMAN      RVTILGHVQRGGTPSAFDRILASRMGVEAVIALLEATPDTPACVVSLNGN 350
                             *.*:***:****:* .:****.*:**  *:  ** .  .   * * :..:

1PFK_A|PDBID|CHAIN|SEQUENCE  QLVHHDIIDAIE---NMKRPFKGDWLDCAKKLY--
sp|P08237.2|PFKAM_HUMAN      QAVRLPLME--------------------------
sp|P17858.6|PFKAL_HUMAN      QSVRLPLME--------------------------
sp|Q01813.2|PFKAP_HUMAN      HAVRLPLMECVQMTQDVQKAMDERRFQDAVRLRGR
                             : *:  :::
```

SUPPLEMENTARY TABLE 8B: C-terminal alignment of human PFK-P with EcPFK

Alignment of EcPFK (PDB 1PFK) with C-terminal domains of human PFK-M, PFK-L, and PFK-P isoforms. Residues in human PFK-P (PDB 4XZ2) involved in ligand binding of tetramer interfaces were identified with PISA (http://www.ebi.ac.uk/pdbe/prot_int/pistart.html). Alignments performed with Clustal Omega (https://www.ebi.ac.uk/Tools/msa/clustalo/)

--- Residues involved in Interface 1 and identified in Supplementary Table 3

--- Residues involved in Interface 2 and identified in Supplementary Table 4

--- Active site residues partially buried by ADP or F6P

--- F26BP binding site residues identified in Supplementary Table 6

--- Effector site B (Figure 1 and Supplementary Table 7) highlighting residues from EcPFK (PDB 1pfk) within 4 Å of effector ADP.

--- Effector site A (Figure 1 and Supplementary Table 7) highlighting residues from EcPFK (PDB 1pfk) within 4 Å of effector ADP

```
1PFK_A|PDBID|CHAIN|SEQUENCE  MI------------------------------------------------
sp|P08237.2|PFKAM_HUMAN      CVQVTKDVTKAMDEKKFDEALKLRGRSFMNNWEVYKLLAHVRPPV--SKS
sp|P17858.6|PFKAL_HUMAN      CVQMTKEVQKAMDDKRFDEATQLRGGSFENNWNIYKLLAHQKPPK--EKS
sp|Q01813.2|PFKAP_HUMAN      --------------------------SFAGNLNTYKRLAIKLPDDQIPKT 409


1PFK_A|PDBID|CHAIN|SEQUENCE  --KKIGVLTSGGDAPGMNAAIRGVVRSALTEGLEVMGIYDGYLGLYEDRM 49
sp|P08237.2|PFKAM_HUMAN      GSHTVAVMNVGAPAAGMNAAVRSTVRIGLIQGNRVLVVHDGFEGLAKGQI
sp|P17858.6|PFKAL_HUMAN      -NFSLAILNVGAPAAGMNAAVRSAVRTGISHGHTVYVVHDGFEGLAKGQV
sp|Q01813.2|PFKAP_HUMAN      -NCNVAVINVGAPAAGMNAAVRSAVRVGIADGHRMLAIYDGFDGFAKGQI 458
                                .:.::. *.-*.*****:*..** .: .*  :  ::**: *: :.::

1PFK_A|PDBID|CHAIN|SEQUENCE  VQLDRYSVSDMINRGGTFLGSAR-FPEFRDENIRAVAIENLKKRGIDALV 98
sp|P08237.2|PFKAM_HUMAN      EEAGWSYVGGWTGQGGSKLGTKRTLPKKSFEQI----SANITKFNIQGLV
sp|P17858.6|PFKAL_HUMAN      QEVGWHDVAGWLGRGGSMLGTKRTLPKGQLESI----VENIRIYGIHALL
sp|Q01813.2|PFKAP_HUMAN      KEIGWTDVGGWTGQGGSILGTKRVLPGKYLEEI----ATQMRTHSINALL 504
                              : .   *..  .:**:-**:-* :*    *.*      ::   .*..*:

1PFK_A|PDBID|CHAIN|SEQUENCE  VIGGDGSYMGAMRLT-------EMGFPCIGLPGTIDNDIKGTDYTIGFFT 141
sp|P08237.2|PFKAM_HUMAN      IIGGFEAYTGGLELMEGRKQFDELCIPFVVIPATVSNNVPGSDFSVGADT
sp|P17858.6|PFKAL_HUMAN      VVGGFEAYEGVLQLVEARGRYEELCIVMCVIPATISNNVPGTDFSLGSDT
sp|Q01813.2|PFKAP_HUMAN      IIGGFEAYLGLLELSAAREKHEEFCVPMVMVPATVSNNVPGSDFSIGADT 554
```

```
                             ::**-- :* * :.*        *: .    :*.*:.*:: *:*:::*  *

1PFK_A|PDBID|CHAIN|SEQUENCE  ALSTVVEAIDRLRDTSS-SHQRISVVEVMGRYCGDLTLAAAIAGGCEFVV 190
sp|P08237.2|PFKAM_HUMAN      ALNTICTTCDRIKQSAAGTKRRVFIIETMGGYCGYLATMAGLAAGADAAY
sp|P17858.6|PFKAL_HUMAN      AVNAAMESCDRIKQSASGTKRRVFIVETMGGYCGYLATVTGIAVGADAAY
sp|Q01813.2|PFKAP_HUMAN      ALNTITDTCDRIKQSASGTKRRVFIIETMGGYCGYLANMGGLAAGADAAY 604
                             *:.:   : *:::::::-:::*:-::*.**-*** *:   .:* *.: .

1PFK_A|PDBID|CHAIN|SEQUENCE  VPEVEFSREDLVNEIKAGIAKGKK---HAIVAITEHMCDVDELAHFIE-- 235
sp|P08237.2|PFKAM_HUMAN      IFEEPFTIRDLQANVEHLVQKMKTTVKRGLV-LRNEKCNENYTTDFIFNL
sp|P17858.6|PFKAL_HUMAN      VFEDPFNIHDLKVNVEHMTEKMKTDIQRGLV-LRNEKCHDYYTTEFLYNL
sp|Q01813.2|PFKAP_HUMAN      IFEEPFDIRDLQSNVEHLTEKMKTTIQRGLV-LRNESCSENYTTDFIYQL 653
                             : *  *--.**- :::    * *.   :.:* : :. *-----:.*:---

1PFK_A|PDBID|CHAIN|SEQUENCE  -KETG---RETRATVLGHIQRGGSPVPYDRILASRMGAYAIDLLLAGY-- 279
sp|P08237.2|PFKAM_HUMAN      YSEEGKGIFDSRKNVLGHMQQGGSPTPFDRNFATKMGAKAMNWMSGKIKE
sp|P17858.6|PFKAL_HUMAN      YSSEGKGVFDCRTNVLGHLQQGGAPTPFDRNYGTKLGVKAMLWLSEKLRE
sp|Q01813.2|PFKAP_HUMAN      YSEEGKGVFDCRKNVLGHMQQGGAPSPFDRNFGTKISARAMEWITAKLKE 703
                              ..-*-   : *  .****:*:**:* *:**  .:::.. *:  :

1PFK_A|PDBID|CHAIN|SEQUENCE  -------------GGRCVGIQNEQLVHHDIIDAIE---NM-KRPFKGDWL 312
sp|P08237.2|PFKAM_HUMAN      SYRNGRIFANTPDSGCVLGMRKRALVFQPVAELKDQTDFEHRIPKEQWWL
sp|P17858.6|PFKAL_HUMAN      VYRKGRVFANAPDSACVIGLKKKAVAFSPVTELKKDTDFEHRMPREQWWL
sp|Q01813.2|PFKAP_HUMAN      ARGRGKKFT-TDDSICVLGISKRNVIFQPVAELKKQTDFEHRIPKEQWWL 752
                                          .   :*: :. : .  : :  .      : * :  **

1PFK_A|PDBID|CHAIN|SEQUENCE  DCAKKLY-------------------------------
sp|P08237.2|PFKAM_HUMAN      KLRPILKILAKYEIDLDTSDHAHLEHITRKRS-GEAAV
sp|P17858.6|PFKAL_HUMAN      SLRLMLKMLAQYRISMAAYVSGELEHVTRRTLSMDKGF
sp|Q01813.2|PFKAP_HUMAN      KLRPLMKILAKYKASYDVSDSGQLEHVQPWS------V
                             .    :
```

SUPPLEMENTARY TABLE 9A

Pearson correlation co-efficients for important glycolytic enzymes in non-cancerous tissues

	HK1	HK2	PFK-M	PFK-L	PFK-P	PFKFB3	PKM	ALDO-A	ALDO-B	ALDO-C	ENO-1	ENO-2	ENO-3	G6PD	GAPDH	LDH-A	LDH-B	LDH-C	PGAM-1	PGAM-2	PGI	PGK-1	TPI
**HK1**		-1.43E-01	1.99E-01	-7.54E-02	1.19E-01	-1.18E-01	2.56E-01	1.64E-01	-4.25E-01	2.34E-01	2.19E-01	1.90E-01	-1.51E-02	2.77E-01	2.63E-01	4.87E-03	2.07E-01	-1.79E-01	1.79E-01	1.53E-01	2.76E-01	1.18E-01	3.56E-01
**HK2**	-1.43E-01		-3.24E-01	-5.95E-02	-5.35E-02	5.28E-01	2.61E-02	-1.63E-01	-2.04E-01	-1.47E-02	8.22E-02	1.37E-01	-2.28E-01	2.95E-01	-1.44E-01	9.42E-02	-1.17E-01	7.12E-02	-1.36E-01	-2.09E-01	1.29E-02	1.61E-01	3.39E-02
**PFK-M**	1.99E-01	-3.24E-01		1.36E-01	-1.58E-01	1.52E-01	7.04E-02	7.75E-01	-1.62E-01	1.83E-01	-1.40E-01	5.31E-02	8.86E-01	2.31E-01	1.71E-01	-1.47E-01	8.29E-02	-1.81E-02	8.25E-02	9.33E-01	3.58E-01	-1.11E-01	2.48E-01
**PFK-L**	-7.54E-02	-5.95E-02	1.36E-01		6.79E-01	1.39E-02	4.81E-01	5.78E-01	5.38E-02	3.07E-02	5.81E-01	1.39E-01	2.40E-01	6.36E-01	4.64E-01	1.25E-01	-3.88E-01	-9.85E-02	4.55E-01	2.98E-01	5.97E-01	3.61E-01	4.95E-01
**PFK-P**	1.19E-01	-5.35E-02	-1.58E-01	6.79E-01		-2.86E-01	6.66E-01	1.24E-01	-3.20E-02	4.83E-02	7.39E-01	2.31E-01	-1.97E-01	4.69E-01	5.14E-01	1.01E-01	-4.43E-02	-6.55E-02	5.60E-01	-1.57E-01	6.91E-01	3.49E-01	5.20E-01
**PFKFB3**	-1.18E-01	5.28E-01	1.52E-01	1.39E-02	-2.86E-01		-1.81E-01	1.06E-01	-1.38E-01	2.40E-01	-2.25E-01	2.09E-01	2.47E-01	2.60E-02	-2.29E-01	-2.16E-01	-9.19E-02	-9.26E-02	-2.44E-01	1.87E-01	-8.47E-02	-5.68E-02	-2.22E-01
**PKM**	2.56E-01	2.61E-02	7.04E-02	4.81E-01	6.66E-01	-1.81E-01		4.37E-01	-2.10E-01	2.12E-03	9.05E-01	1.99E-01	7.33E-02	6.20E-01	8.73E-01	5.28E-01	6.78E-02	-1.39E-01	8.63E-01	4.30E-02	7.84E-01	6.10E-01	8.15E-01
**ALDO-A**	1.64E-01	-1.63E-01	7.75E-01	5.78E-01	1.24E-01	1.06E-01	4.37E-01		-2.09E-01	-3.53E-02	3.18E-01	-2.11E-02	8.39E-01	5.89E-01	5.30E-01	1.87E-01	-1.93E-01	-6.99E-02	4.04E-01	8.61E-01	5.76E-01	3.05E-01	5.40E-01
**ALDO-B**	-4.25E-01	-2.04E-01	-1.62E-01	5.38E-02	-3.20E-02	-1.38E-01	-2.10E-01	-2.09E-01		-6.98E-02	-1.41E-01	-1.12E-01	-1.19E-01	-1.35E-01	-2.07E-01	-1.49E-01	-2.68E-02	-5.95E-02	-2.17E-01	-1.32E-01	-1.17E-01	-2.53E-01	-2.69E-01
**ALDO-C**	2.34E-01	-1.47E-02	1.83E-01	3.07E-02	4.83E-02	2.40E-01	2.12E-03	-3.53E-02	-6.98E-02		-3.65E-02	9.20E-01	-7.39E-02	1.39E-01	-7.59E-02	-2.68E-01	5.93E-02	-8.95E-02	1.25E-01	5.36E-04	2.56E-01	-2.77E-01	1.33E-01
**ENO-1**	2.19E-01	8.22E-02	-1.40E-01	5.81E-01	7.39E-01	-2.25E-01	9.05E-01	3.18E-01	-1.41E-01	-3.65E-02		2.04E-01	-7.61E-02	6.70E-01	7.61E-01	5.09E-01	-2.60E-02	-1.90E-01	7.60E-01	-9.81E-02	7.30E-01	5.61E-01	7.18E-01
**ENO-2**	1.90E-01	1.37E-01	5.31E-02	1.39E-01	2.31E-01	2.09E-01	1.99E-01	-2.11E-02	-1.12E-01	9.20E-01	2.04E-01		-1.05E-01	3.44E-01	1.64E-02	-2.40E-01	-1.26E-01	-5.56E-02	2.14E-01	-9.58E-02	3.78E-01	-2.17E-01	2.10E-01
**ENO-3**	-1.51E-02	-2.28E-01	8.86E-01	2.40E-01	-1.97E-01	2.47E-01	7.33E-02	8.39E-01	-1.19E-01	-7.39E-02	-7.61E-02	-1.05E-01		2.67E-01	1.25E-01	-1.32E-01	-2.21E-01	-4.29E-02	9.60E-03	9.14E-01	2.57E-01	-1.24E-01	1.34E-01
**G6PD**	2.77E-01	2.95E-01	2.31E-01	6.36E-01	4.69E-01	2.60E-02	6.20E-01	5.89E-01	-1.35E-01	1.39E-01	6.70E-01	3.44E-01	2.67E-01		4.55E-01	1.30E-01	-2.93E-01	-7.96E-02	3.99E-01	3.81E-01	7.27E-01	2.92E-01	6.50E-01
**GAPDH**	2.63E-01	-1.44E-01	1.71E-01	4.64E-01	5.14E-01	-2.29E-01	8.73E-01	5.30E-01	-2.07E-01	-7.59E-02	7.61E-01	1.64E-02	1.25E-01	4.55E-01		6.46E-01	2.27E-01	-1.59E-01	8.63E-01	1.46E-01	6.80E-01	8.19E-01	8.60E-01
**LDH-A**	4.87E-03	9.42E-02	-1.47E-01	1.25E-01	1.01E-01	-2.16E-01	5.28E-01	1.87E-01	-1.49E-01	-2.68E-01	5.09E-01	-2.40E-01	-1.32E-01	1.30E-01	6.46E-01		3.54E-01	-7.58E-02	6.01E-01	-1.53E-01	2.86E-01	6.64E-01	5.52E-01
**LDH-B**	2.07E-01	-1.17E-01	8.29E-02	-3.88E-01	-4.43E-02	-9.19E-02	6.78E-02	-1.93E-01	-2.68E-02	5.93E-02	-2.60E-02	-1.26E-01	-2.21E-01	-2.93E-01	2.27E-01	3.54E-01		-1.39E-01	2.44E-01	-1.01E-01	1.07E-01	3.02E-01	1.43E-01
**LDH-C**	-1.79E-01	7.12E-02	-1.81E-02	-9.85E-02	-6.55E-02	-9.26E-02	-1.39E-01	-6.99E-02	-5.95E-02	-8.95E-02	-1.90E-01	-5.56E-02	-4.29E-02	-7.96E-02	-1.59E-01	-7.58E-02	-1.39E-01		-2.15E-01	-2.04E-02	-1.65E-01	-2.33E-02	-1.90E-01
**PGAM-1**	1.79E-01	-1.36E-01	8.25E-02	4.55E-01	5.60E-01	-2.44E-01	8.63E-01	4.04E-01	-2.17E-01	1.25E-01	7.60E-01	2.14E-01	9.60E-03	3.99E-01	8.63E-01	6.01E-01	2.44E-01	-2.15E-01		1.99E-02	6.76E-01	6.74E-01	7.61E-01
**PGAM-2**	1.53E-01	-2.09E-01	9.33E-01	2.98E-01	-1.57E-01	1.87E-01	4.30E-02	8.61E-01	-1.32E-01	5.36E-04	-9.81E-02	-9.58E-02	9.14E-01	3.81E-01	1.46E-01	-1.53E-01	-1.01E-01	-2.04E-02	1.99E-02		3.10E-01	-2.93E-02	2.27E-01
**PGI**	2.76E-01	1.29E-02	3.58E-01	5.97E-01	6.91E-01	-8.47E-02	7.84E-01	5.76E-01	-1.17E-01	2.56E-01	7.30E-01	3.78E-01	2.57E-01	7.27E-01	6.80E-01	2.86E-01	1.07E-01	-1.65E-01	6.76E-01	3.10E-01		3.58E-01	7.65E-01
**PGK-1**	1.18E-01	1.61E-01	-1.11E-01	3.61E-01	3.49E-01	-5.68E-02	6.10E-01	3.05E-01	-2.53E-01	-2.77E-01	5.61E-01	-2.17E-01	-1.24E-01	2.92E-01	8.19E-01	6.64E-01	3.02E-01	-2.33E-02	6.74E-01	-2.93E-02	3.58E-01		6.33E-01
**TPI**	3.56E-01	3.39E-02	2.48E-01	4.95E-01	5.20E-01	-2.22E-01	8.15E-01	5.40E-01	-2.69E-01	1.33E-01	7.18E-01	2.10E-01	1.34E-01	6.50E-01	8.60E-01	5.52E-01	1.43E-01	-1.90E-01	7.61E-01	2.27E-01	7.65E-01	6.33E-01	

SUPPLEMENTARY TABLE 9B

Pearson correlation co-efficients for important glycolytic enzymes in cancerous tissues

	HK1	HK2	PFK-M	PFK-L	PFK-P	PFKFB3	PKM	ALDO-A	ALDO-B	ALDO-C	ENO-1	ENO-2	ENO-3	G6PD	GAPDH	LDH-A	LDH-B	LDH-C	PGAM-1	PGAM-2	PGI	PGK-1	TPI
**HK1**		1.36E-01	-1.06E-01	6.62E-02	2.19E-01	1.18E-01	1.97E-01	4.05E-01	-1.06E-01	3.88E-01	1.66E-01	1.02E-01	-7.93E-02	-8.26E-02	-5.23E-03	1.98E-01	1.08E-01	-9.70E-04	1.99E-01	-4.76E-02	2.01E-01	2.05E-01	1.54E-01
**HK2**	1.36E-01		-1.19E-01	4.27E-01	-1.24E-01	1.71E-01	-1.80E-01	1.16E-01	-6.95E-02	2.78E-01	1.60E-01	4.74E-02	-2.09E-01	-4.06E-01	-1.31E-01	-9.44E-02	3.90E-02	1.32E-01	3.53E-02	8.73E-03	-1.23E-01	1.89E-02	1.49E-01
**PFK-M**	-1.06E-01	-1.19E-01		-1.62E-01	-1.90E-02	-1.71E-01	-1.61E-01	-2.13E-01	-3.40E-02	1.35E-01	-1.49E-01	5.56E-01	5.51E-01	-3.87E-02	3.80E-01	4.77E-02	3.35E-01	1.79E-02	2.32E-01	-8.74E-02	3.45E-01	1.35E-01	1.17E-01
**PFK-L**	6.62E-02	4.27E-01	-1.62E-01		1.44E-01	3.67E-02	1.08E-01	1.85E-01	1.02E-02	2.44E-02	3.71E-01	-2.48E-01	-1.83E-01	-2.41E-01	5.91E-02	2.90E-01	2.81E-01	8.46E-02	5.22E-01	-5.11E-02	-5.83E-02	2.86E-01	3.56E-01
**PFK-P**	2.19E-01	-1.24E-01	-1.90E-02	1.44E-01		3.55E-01	7.26E-01	4.26E-01	-8.80E-02	-1.29E-01	2.54E-01	-1.40E-02	-6.07E-02	2.23E-01	3.89E-01	4.10E-01	3.95E-02	-1.22E-01	2.90E-01	2.29E-02	5.71E-02	3.52E-01	3.77E-01
**PFKFB3**	1.18E-01	1.71E-01	-1.71E-01	3.67E-02	3.55E-01		2.42E-01	4.95E-02	-7.26E-02	-1.49E-02	-2.21E-02	-1.30E-01	-3.56E-01	-8.45E-02	-5.63E-02	7.22E-02	-1.11E-01	1.82E-01	1.20E-01	6.35E-02	-2.06E-01	-3.43E-02	8.77E-02
**PKM**	1.97E-01	-1.80E-01	-1.61E-01	1.08E-01	7.26E-01	2.42E-01		5.74E-01	-1.15E-01	-1.58E-02	5.45E-01	-1.55E-02	8.63E-02	1.98E-01	5.07E-01	6.02E-01	6.83E-02	2.03E-02	4.27E-01	7.33E-02	2.47E-01	5.86E-01	5.53E-01
**ALDO-A**	4.05E-01	1.16E-01	-2.13E-01	1.85E-01	4.26E-01	4.95E-02	5.74E-01		-1.04E-01	1.78E-01	5.04E-01	-1.37E-02	1.47E-01	3.55E-01	5.00E-01	6.02E-01	3.87E-02	5.18E-02	2.61E-01	9.80E-02	4.49E-01	6.27E-01	5.41E-01
**ALDO-B**	-1.06E-01	-6.95E-02	-3.40E-02	1.02E-02	-8.80E-02	-7.26E-02	-1.15E-01	-1.04E-01		-6.40E-03	-1.11E-01	4.09E-02	-5.06E-02	-7.50E-02	-1.24E-01	-9.11E-02	-1.44E-01	-7.24E-02	-1.30E-01	3.65E-02	1.57E-01	-7.91E-02	-3.58E-02
**ALDO-C**	3.88E-01	2.78E-01	1.35E-01	2.44E-02	-1.29E-01	-1.49E-02	-1.58E-02	1.78E-01	-6.40E-03		2.68E-01	3.14E-01	2.32E-01	-1.64E-01	1.42E-01	1.69E-01	2.99E-01	2.65E-01	1.66E-01	5.92E-02	4.41E-01	4.08E-01	1.58E-01
**ENO-1**	1.66E-01	1.60E-01	-1.49E-01	3.71E-01	2.54E-01	-2.21E-02	5.45E-01	5.04E-01	-1.11E-01	2.68E-01		1.79E-02	2.56E-01	8.87E-02	6.56E-01	6.97E-01	3.75E-01	-6.21E-03	5.14E-01	3.15E-02	3.75E-01	7.83E-01	6.66E-01
**ENO-2**	1.02E-01	4.74E-02	5.56E-01	-2.48E-01	-1.40E-02	-1.30E-01	-1.55E-02	-1.37E-02	4.09E-02	3.14E-01	1.79E-02		4.69E-01	-1.31E-01	2.34E-01	-1.27E-01	2.82E-01	-5.07E-02	1.84E-01	3.11E-02	4.54E-01	1.71E-01	2.17E-01
**ENO-3**	-7.93E-02	-2.09E-01	5.51E-01	-1.83E-01	-6.07E-02	-3.56E-01	8.63E-02	1.47E-01	-5.06E-02	2.32E-01	2.56E-01	4.69E-01		-3.78E-02	5.76E-01	3.95E-01	2.65E-01	-1.03E-01	2.86E-01	-8.23E-02	5.39E-01	5.05E-01	3.66E-01
**G6PD**	-8.26E-02	-4.06E-01	-3.87E-02	-2.41E-01	2.23E-01	-8.45E-02	1.98E-01	3.55E-01	-7.50E-02	-1.64E-01	8.87E-02	-1.31E-01	-3.78E-02		3.36E-01	1.40E-01	-8.85E-02	1.02E-01	-1.97E-01	8.80E-02	1.05E-02	5.78E-02	-6.12E-02
**GAPDH**	-5.23E-03	-1.31E-01	3.80E-01	5.91E-02	3.89E-01	-5.63E-02	5.07E-01	5.00E-01	-1.24E-01	1.42E-01	6.56E-01	2.34E-01	5.76E-01	3.36E-01		6.71E-01	3.82E-01	-3.25E-03	4.83E-01	8.10E-02	5.53E-01	7.94E-01	6.99E-01
**LDH-A**	1.98E-01	-9.44E-02	4.77E-02	2.90E-01	4.10E-01	7.22E-02	6.02E-01	6.02E-01	-9.11E-02	1.69E-01	6.97E-01	-1.27E-01	3.95E-01	1.40E-01	6.71E-01		1.84E-01	5.05E-02	6.19E-01	7.15E-03	4.15E-01	8.12E-01	6.64E-01
**LDH-B**	1.08E-01	3.90E-02	3.35E-01	2.81E-01	3.95E-02	-1.11E-01	6.83E-02	3.87E-02	-1.44E-01	2.99E-01	3.75E-01	2.82E-01	2.65E-01	-8.85E-02	3.82E-01	1.84E-01		-6.23E-02	4.06E-01	-2.13E-02	9.06E-02	4.16E-01	4.22E-01
**LDH-C**	-9.70E-04	1.32E-01	1.79E-02	8.46E-02	-1.22E-01	1.82E-01	2.03E-02	5.18E-02	-7.24E-02	2.65E-01	-6.21E-03	-5.07E-02	-1.03E-01	1.02E-01	-3.25E-03	5.05E-02	-6.23E-02		1.06E-01	3.56E-01	1.04E-01	1.94E-02	-1.02E-01
**PGAM-1**	1.99E-01	3.53E-02	2.32E-01	5.22E-01	2.90E-01	1.20E-01	4.27E-01	2.61E-01	-1.30E-01	1.66E-01	5.14E-01	1.84E-01	2.86E-01	-1.97E-01	4.83E-01	6.19E-01	4.06E-01	1.06E-01		3.30E-02	3.60E-01	6.71E-01	7.24E-01
**PGAM-2**	-4.76E-02	8.73E-03	-8.74E-02	-5.11E-02	2.29E-02	6.35E-02	7.33E-02	9.80E-02	3.65E-02	5.92E-02	3.15E-02	3.11E-02	-8.23E-02	8.80E-02	8.10E-02	7.15E-03	-2.13E-02	3.56E-01	3.30E-02		3.48E-03	5.80E-03	9.16E-03
**PGI**	2.01E-01	-1.23E-01	3.45E-01	-5.83E-02	5.71E-02	-2.06E-01	2.47E-01	4.49E-01	1.57E-01	4.41E-01	3.75E-01	4.54E-01	5.39E-01	1.05E-02	5.53E-01	4.15E-01	9.06E-02	1.04E-01	3.60E-01	3.48E-03		6.17E-01	4.19E-01
**PGK-1**	2.05E-	1.89E-	1.35E-	2.86E-	3.52E-	-3.43E-	5.86E-	6.27E-	-7.91E-	4.08E-	7.83E-	1.71E-	5.05E-	5.78E-	7.94E-	8.12E-	4.16E-	1.94E-	6.71E-	5.80E-	6.17E-		8.14E-

	01	02	01	01	01	02	01	01	02	01	01	01	01	02	01	01	01	02	01	03	01		01
**TPI**	1.54E-01	1.49E-01	1.17E-01	3.56E-01	3.77E-01	8.77E-02	5.53E-01	5.41E-01	-3.58E-02	1.58E-01	6.66E-01	2.17E-01	3.66E-01	-6.12E-02	6.99E-01	6.64E-01	4.22E-01	-1.02E-01	7.24E-01	9.16E-03	4.19E-01	8.14E-01	

SUPPLEMENTARY TABLE 10A

Comparison of K_m^{F6P} from selected publications shows similar absolute values but varying hierarchies for each isoform.

Author	*Year*	*PFK derivation*	*ATP concentration*	*K_mF6P* (µM)		
				PFK-M	*PFK-L*	*PFK-P*
Meienhofer et al(7)	*1980*	*Purified tissue*	*500*µM	*1800*	*1400*	*620*
Dunaway et al(8)	*1988*	*Partially purified tissue*	*1000*µM	*2000*	*3500-4000*	*350-550*
Sanchez-Martinez et al(9)	*2000*	*Recombinant (yeast)*	*5000*µM	*2000*	*-*	*3900 (ascitic tumour cell)*
Moreno-Sanchez et al(10)	*2012*	*Tissue supernatant*	*550-700*µM	*145 (rat heart)*	*3000 (rat liver)*	*1100 (HeLa cell)*
Fernandes et al (this study)	*2020*	*Recombinant*	*500*µM	*147*	*1360*	*1333*

SUPPLEMENTARY TABLE 10B

Comparison of K_m^{ATP} from selected publications shows similar absolute values but varying hierarchies for each isoform.

Author	*Year*	*PFK derivation*	*F6P concentration*	*K_mATP* (µM)		
				PFK-M	*PFK-L*	*PFK-P*
Sanchez-Martinez et al(9)	*2000*	*Recombinant (yeast)*	*2000*µM	*600*	*-*	*40 (ascitic tumour cell)*
Moreno-Sanchez et al(10)	*2012*	*Tissue supernatant*	*8600*µM	*74 (rat heart)*	*29 (rat liver)*	*39.5 (HeLa cell)*
Fernandes et al (this study)	*2020*	*Recombinant*	*4000*µM	*151*	*160*	*275*

REFERENCES

1. Traut TW. Physiological concentrations of purines and pyrimidines. Molecular and Cellular Biochemistry. 1994;140(1):1–22.
2. Fersht. Structure and Mechanism in Protein Science. 2nd ed. WSPC; 1999. 366 p.
3. Lawson JWR, Uyeda K. Effects of Insulin and Work on Fructose 2 , 6 Bisphosphate Content and Phosphofructokinase Activity in Perfused Rat Hearts. Journal of Biological Chemistry. 1987;262(7):3165–73.
4. Kauppinen R, Hiltunen JK, Hassinen IE. Compartmentation of citrate in relation to the regulation of glycolysis and the mitochondrial transmembrane proton electrochemical potential gradient in isolated perfused rat heart. Biochimica et biophysica acta. 1982;681:286–91.
5. Albe KR, Butler MH, Wright BE. Cellular concentrations of enzymes and their substrates. Journal of Theoretical Biology. 1990 Mar;143(2):163–95.
6. Kjerulf-Jensen K. The Phosphate Esters Formed in the Liver Tissue of Rats and Rabbits during Assimilation of Hexoses and Glycerol.1. Acta Physiologica Scandinavica. 1942 Aug;4(3-4):249–58.
7. Meienhofer MC, Cottreau D, Dreyfus JC, Kahn A. Kinetic properties of human F4 phosphofructokinase. FEBS Letters. 1980;110(2):219–22.
8. Dunaway G, Kasten TP, Sebo T, Trapp R. Analysis of the phosphofructokinase subunits and isoenzymes in human tissues. The Biochemical Journal. 1988 May;251(3):677–83.
9. Sanchez-Martinez C, Estevez AM, Aragón JJ. Phosphofructokinase C Isozyme from Ascites Tumor Cells: Cloning, Expression, and Properties. Biochemical and Biophysical Research Communications. 2000;640:635–40.
10. Moreno-Sánchez R, Marin-Hernandez A, Gallardo-Perez JC, Quezada H, Encalada R, Rodriguez-Enriquez S, et al. Phosphofructokinase Type 1 Kinetics, Isoform Expression, and Gene Polymorphisms in Cancer Cells. Journal of Cellular Biochemistry. 2012;113(5):1692–703.
